# Global Analysis of Genetic, Epigenetic and Transcriptional Polymorphisms in *Arabidopsis thaliana* Using Whole Genome Tiling Arrays

**DOI:** 10.1371/journal.pgen.1000032

**Published:** 2008-03-21

**Authors:** Xu Zhang, Shinhan Shiu, Andrew Cal, Justin O. Borevitz

**Affiliations:** 1 Department of Ecology and Evolution, University of Chicago, Chicago, Illinois, United States of America; 2 Department of Plant Biology, Michigan State University, East Lansing, Michigan, United States of America; 3 Department of Molecular Genetics and Cell Biology, University of Chicago, Chicago, Illinois, United States of America; The University of North Carolina at Chapel Hill, United States of America

## Abstract

Whole genome tiling arrays provide a high resolution platform for profiling of genetic, epigenetic, and gene expression polymorphisms. In this study we surveyed natural genomic variation in cytosine methylation among *Arabidopsis thaliana* wild accessions Columbia (Col) and Vancouver (Van) by comparing hybridization intensity difference between genomic DNA digested with either methylation-sensitive (*Hpa*II) or -insensitive (*Msp*I) restriction enzyme. Single Feature Polymorphisms (SFPs) were assayed on a full set of 1,683,620 unique features of Arabidopsis Tiling Array 1.0F (Affymetrix), while constitutive and polymorphic CG methylation were assayed on a subset of 54,519 features, which contain a 5′CCGG3′ restriction site. 138,552 SFPs (1% FDR) were identified across enzyme treatments, which preferentially accumulated in pericentromeric regions. Our study also demonstrates that at least 8% of all analyzed CCGG sites were constitutively methylated across the two strains, while about 10% of all analyzed CCGG sites were differentially methylated between the two strains. Within euchromatin arms, both constitutive and polymorphic CG methylation accumulated in central regions of genes but under-represented toward the 5′ and 3′ ends of the coding sequences. Nevertheless, polymorphic methylation occurred much more frequently in gene ends than constitutive methylation. Inheritance of methylation polymorphisms in reciprocal F1 hybrids was predominantly additive, with F1 plants generally showing levels of methylation intermediate between the parents. By comparing gene expression profiles, using matched tissue samples, we found that magnitude of methylation polymorphism immediately upstream or downstream of the gene was inversely correlated with the degree of expression variation for that gene. In contrast, methylation polymorphism within genic region showed weak positive correlation with expression variation. Our results demonstrated extensive genetic and epigenetic polymorphisms between Arabidopsis accessions and suggested a possible relationship between natural CG methylation variation and gene expression variation.

## Author Summary

The functional expression of DNA sequence depends on the chromatin status. Epigenetic marks at specific loci could affect local chromatin accessibility, thus affect the gene activity of that loci. We applied an enzyme methylome approach to globally detect one type of epigenetic mark, cytosine methylation at CCGG restriction sites. Simultaneous transcriptional profiling allowed gene expression differences to be compared with DNA methylation differences, suggesting functional regulatory regions. Our method reveals natural variation in chromatin patterns which may underlie phenotypic variation.

## Introduction

Epigenetic modification has a profound effect on genome activity. In eukaryotes, DNA methylation of cytosine residues is a common phenomenon [1] that serves as a mechanism to suppress mobile elements [2],[3] and other nuclear processes such as transcription and recombination [4]. Globally, DNA methylation is closely associated with histone modification and other aspects of chromatin status [5]. DNA methylation within promoter regions can inhibit binding of transcription factors [6] or recruit methyl-CG binding proteins which repress transcription initiation [7]; thus regulates an intrinsic component of growth and development [8],[9]. Exceedingly dense methylation in intra-genic regions silences transcription by reducing Pol II elongation efficiency [10],[11].

Evidence of DNA methylation regulating gene expression has accumulated from the study of several epigenetic mutants, or epimutants, such as *fwa* [12] and *superman* [13] in *Arabidopsis thaliana* and *agouti* [14] in mouse. In these epimutants, affected genes exhibit unusual DNA methylation within promoter regions [12]–[14]. Recent genome-wide analysis of methylation mutants using tiling arrays uncovered the ubiquitous up-regulation of gene expression in hypomethylated regions, especially for pseudogenes and transposons [15],[16]. It remains unclear, however, how gene expression is regulated by DNA methylation, and specifically how epigenetic polymorphisms contribute to gene expression variation in a natural context.

Patterns and inheritance of DNA methylation are substantially different between mammals and plants. In mammals, DNA methylation mostly occurs at CG sites and the whole genome is densely methylated except for CpG islands [17],[18]. Meiotic inheritance of DNA methylation in mammals is rare [14]. In plants, non-CG methylation at CNG and CNN sites also exist and methylation in plant genomes is relatively sparse outside of heterochromatin [15],[16]. Meiotic inheritance of DNA methylation is frequently observed in plants [19],[20]. Several recent studies applied anti-5methylcytosine Chromatin Immuno-Precipitation followed by array hybridization (ChIP-chip) and assessed the global patterns of constitutive methylation in *A. thaliana* [15],[16]. These studies indicate a significant proportion of DNA methylation in genic regions. Very recently, Vaughn and coworkers reported the study of natural epigenetic variation between *A. thaliana* Col and Landsberg (Ler) accessions using a methylation-dependent McrBC enzyme digestion approach to profile the entire chromosome 4 at a resolution of 1 kb [21]. They found that DNA methylation was highly polymorphic among Arabidopsis strains but that DNA methylation in euchromatin regions had little observable effect on gene expression.

In this study, we conducted methyl-sensitive and -insensitive enzyme digestion of genomic DNA from two Arabidopsis accessions, Col and Van, as well as their reciprocal F1 hybrids, followed by hybridization to the Arabidopsis tiling 1.0F array [16], which tiles the whole genome with ∼1.7×10^6^ unique array features at 35bp resolution. This approach allows us to precisely locate the genome positions of both constitutive and polymorphic CG methylation, using ∼55,000 CCGG-containing features interrogating about half of all CCGG sites of the entire Arabidopsis genome. As this approach preserves the majority of genomic hybridization signals, SFPs can be assessed simultaneously [22]. Furthermore, we compared the methylation and gene expression profiles derived from the same biological samples. Our results demonstrated extensive genetic and epigenetic polymorphisms between natural accessions and a predominantly additive inheritance of CG methylation polymorphisms. Our results also suggested possible contribution of natural CG methylation polymorphisms to gene expression variation. The enzyme methylome approach we present here could be extended to several other isoschizomer pairs such as *Sau3A*I/*Mbo*I for a more complete analysis.

## Results

### Genetic Variation of Arabidopsis Natural Accessions

**Figure 1:**
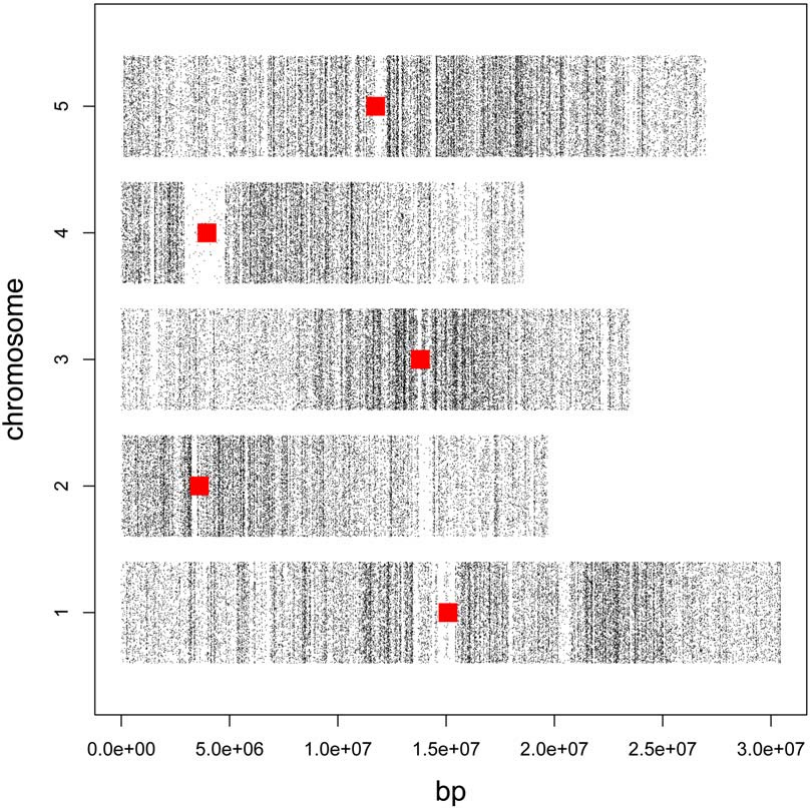
Genomic Distribution of SFPs. The base positions (x-axis) for 120,810 SFPs (FDR 1%) with greater Col intensity were plotted along chromosomes (y-axis). Red bars indicated the positions of BAC clones for centromere sequences (http://www.ncbi.nlm.nih.gov/mapview).

The Arabidopsis Tiling 1.0F array (Affymetrix) contains 1,683,620 unique features, which allowed us to survey SFPs between Col and Van accessions at a near saturating resolution. For each genotype, genomic DNA samples from 4 biological replicates were digested with either *Hpa*II or *Msp*I. The differential enzyme digestion can be regarded as pseudo-technical replicates; therefore provided additional detection power. At 1% false discover rate (FDR), 138,552 features exhibited significant hybridization differences between accessions. Among them, the Van genotype had a greater signal for 17,742 features and the Col genotype showed a greater signal for 120,810 features (Table S1A). As the array features were designed from Col genome sequence, SFPs with greater signal in Col suggest sequence polymorphisms ranging from Single Nucleotide Polymorphisms (SNPs) to complete deletion of the loci in Van. Features with greater signals in Van are likely due to sequence duplications or represent cross hybridization from regions deleted in Col; thus, the exact genome position of these features is unclear. Therefore these features were removed prior to analysis of genome distribution of SFPs. All SFPs were excluded from transcription analysis described below. Similar to recent reports [23],[24], more SFPs occurred in pericentromeric regions than in euchromatin arms (Figure 1). To assess the genic distribution of SFPs, we calculated the frequencies of SFPs for several annotation categories (TableS1B). As expected, the frequency of SFPs was higher within inter-genic regions than within coding sequences (χ^2^ = 7660, p-value <2.2e-16).

### Constitutive and Polymorphic CG Methylation

We then focused on 54,519 CCGG-containing features, which interrogate about half of the ∼130,000 CCGG sites in the genome, for methylation analysis. These features span the whole genome baring a slight under-representation in the centromeric regions (Figure S1A). Restriction enzymes *Hpa*II and *Msp*I both recognize the CCGG sequence, but *Hpa*II digestion is inhibited by methylation at the internal cytosine while *Msp*I is not. A significantly greater hybridization signal at the target feature in the *Hpa*II sample suggests that the locus is preferentially cleaved by *Msp*I, indicating a quantitative change in methylation of the underlying genomic DNA. For each CCGG-containing feature, we modeled hybridization intensity by testing genotype and enzyme main effects and a genotype×enzyme interaction effect. The genotype effect contrasts two genotypes across enzyme treatments and detects SFPs. The enzyme effect contrasts enzyme treatments across genotypes and detects constitutive CG methylation (consistent between Col and Van) as features with significantly greater signal in *Hpa*II sample than in *Msp*I sample. The genotype×enzyme interaction effect compares differential enzyme responses between genotypes, which are CG methylation polymorphisms. For each effect, we calculated a nominal p-value based on 1000 permutations. A total of 4,522 features with greater *Hpa*II signal were significant (p<0.05) for enzyme effect (Table 1). We also observed features with a greater signal in *Msp*I sample than in *Hpa*II sample, which was likely due to the conservative quantile normalization procedure. There were 5,215 features significant (p<0.05) for genotype×enzyme interaction: 3,700 corresponding to Col-specific methylation and 1,515 corresponding to Van-specific methylation (Table 1). For this enzyme methylome approach fragment size variation after enzyme digestion could potentially cause variation in labeling. Furthermore, relative position of the CCGG sequence within a feature could affect the detection sensitivity. Evaluation of these aspects, however, demonstrated that the fragment size variation (Figure S1B) as well as the relative position of CCGG sequence within feature (Figure S1C) did not significantly affect the detection of constitutive or polymorphic CG methylation.

**Table 1:** Summary of Constitutive and Polymorphic CG Methylation Sites.

Constitutive CG methylation		Polymorphic CG methylation		
p-value	Counts^a^	p-value	Col-specific^b^	Van-specific^b^
<0.01	2373	<0.01	1062	407
<0.03	3583	<0.03	2389	944
<0.05	4522	<0.05	3700	1515
Gene^c^	3448 (17%)	Gene^c^	3954 (19%)	
Total gene^d^	20609	Total gene^d^	20609	
Promoter^e^	176 (5%)	Promoter^e^	432 (13%)	
Total promoter^f^	3246	Total promoter^f^	3246	
Intergenic^g^	877 (11%)	Intergenic^g^	775 (10%)	
Total intergenic^h^	8276	Total intergenic^h^	8276	

a The number of significant constitutive CG methylation sites at different p-value thresholds.

b The number of significant Col-specific or Van-specific CG methylation sites at different p-value thresholds,

c The number of annotated gene sequences with feature(s) significant (p<0.05) for enzyme effect or for genotype×enzyme interaction.

d The number of annotated gene sequences with CCGG-containing feature(s).

e The number of promoters with feature(s) significant (p<0.05) for enzyme effect or for genotype×enzyme interaction. Promoters were defined as sequences from transcriptional start to 500 bp upstream.

f The number of promoters with CCGG-containing feature(s).

g The number of inter-genic features (not within annotated gene sequences or promoters) significant (p<0.05) for enzyme effect or for genotype×enzyme interaction.

h The number of inter-genic CCGG-containing features.

To independently validate our tiling array results, we evaluated the false discovery rate (FDR) of our methylation polymorphism calls by PCR. Seedlings from the same maternal seed batches (Materials and Methods) were grown to the same developmental stage under the same growth condition as in the microarray experiments. Genomic DNA from three independent maternal seed batch replicates was used for each genotype. We randomly selected 41 loci from 3,333 features with significant (p<0.03) genotype×enzyme interaction. Genomic PCR following differential restriction digest confirmed 24/24 loci as Col-specific methylation (Figure S2A) and 17/17 loci as Van-specific methylation (Figure S2B). The confirmation of all 41 loci, however, suggested that our permutation based false positive rate threshold at p<0.03 was perhaps overly conservative, thus missing many true positives. For a rough estimation of the false negative rate, we randomly selected 33 loci from all 54,519 CCGG-containing features. Genomic PCR indicated 4/33 as constitutive CG methylation and 3/33 as methylation polymorphisms (Figure S2C). By extension, ∼12% or ∼7,000 features could contain constitutive methylation site and ∼9% or ∼5,000 features would contain methylation polymorphism. Accordingly, we identified 4,522 features of enzyme effect and 5,215 features of genotype×enzyme interaction at p<0.05 for further analysis to balance the false positive and false negative rate. The 54,519 CCGG features analyzed covered 20,609 genes and 3,246 promoters (defined as transcriptional start site to 500 bp upstream). We found that 17% of genes but only 5% of promoters were methylated in both genotypes (Table 1). Enrichment for genic methylation over regulatory methylation agrees with other recent studies [15],[16],[21]. About 19% of genes and notably 13% of promoters contained methylation polymorphism (Table 1).

As this enzyme methylome approach is site-specific, we evaluated the overall cytosine methylation pattern surrounding the detected polymorphic loci by quantitative measurements. Using bisulfite-treated genomic DNA, we typed (see epityper in Materials and Methods) 2 regions and sequenced 3 regions spanning 5 loci detected polymorphic for specific CCGG methylation. The epityper experiment quantified the methylation level for all CG sites within ∼300 bp across three independent maternal seed batch replicates for each genotype. In the bisulfite sequencing, we calculated the percent methylation for all cytosine residues within ∼150 bp for a single maternal seed batch for each genotype. All of the 5 polymorphic sites detected by microarray were confirmed by these methods (Figure S3, Table S2). Interestingly, the status of CG methylation across the same segment showed a great degree of heterogeneousness, ranging from 0 to 100% methylation (Figure S3). The level of polymorphism within the same segment was also variable; some CG sites were polymorphic while others were not. Nevertheless, within the same segment sites that were polymorphic seen to be in phase with either Col or Van showing enriched methylation (Figure S3). Thus the polymorphic sites detected by this enzyme methylome approach in part reflect the local status of methylation variation but also show unique variation. Consistent with a previous report [25], the majority of non CG sites were not methylated within gene regions.

We further compared the constitutive methylation sites detected by our method with two recently published results using ChIP-chip method in *A. thaliana* [15],[16]. Comparison with data performed on the same microarray platform [16] showed that 46% of the constitutive CCGG sites detected here were within the methylated regions detected by ChIP-chip (Table S3). The overlap of the two methods was significant (χ^2^ = 107050, p<2.2e-16; Table S3). The remaining 54% of CCGG sites within ChIP-chip regions that were not detected by our method are likely due to different statistical thresholds, truly unmethylated CCGG sites within methylated regions, and/or due to the difference of the biological samples (developmental stages and growth conditions) used in these studies. Furthermore, 73% of constitutive methylation sites detected in our study were outside of the methylated regions detected by ChIP-chip (Table S3). In fact, among the 6 loci validated by quantitative method (5 polymorphic and 1 constitutive sites), 5 of them were outside of the ChIP-chip regions (Figure S3), implying that immuno-precipitation by anti-5methylcytosine used in ChIP-chip may depend on relative dense regional methylation. Comparison with the ChIP-chip method using a different microarray platform [15] led to a similar conclusion (Table S3). The methylated CCGG sites detected by our method showed a slightly higher frequency in larger ChIP-chip segments, in comparison with unmethylated CCGG sites (Figure S4).

### Genomic and Genic Distribution of Constitutive and Polymorphic CG Methylation

**Figure 2:**
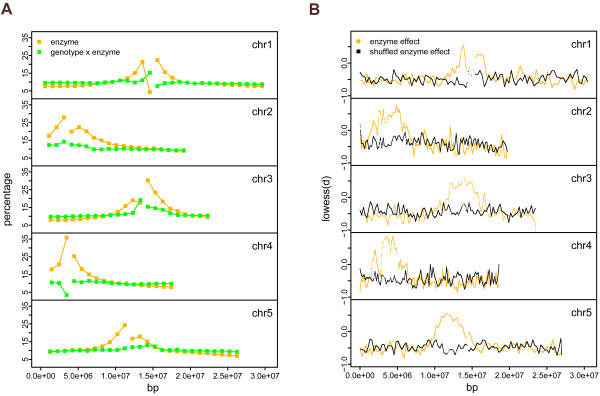
Genomic Distribution of Constitutive and Polymorphic CG Methylation Sites. (A) Percent constitutive (orange) or polymorphic (green) CG methylation sites (y-axis) along chromosomes. The x-axis indicated the chromosome positions in bp. The gaps on each chromosome indicated the positions of BAC clones for centromere sequences. Each chromosome was divided into 1 Mb bins starting from the ends of the centromere gap toward chromosome arms. For each bin, percent constitutive or polymorphic CG methylation sites was calculated as the number of features containing constitutive or polymorphic CG methylation divided by the number of CCGG-containing features. (B) Co-methylation along chromosomes. The d scores of enzyme effect for 54,519 CCGG-containing features were Lowess-smoothed with a window size of 200 kb (orange), or were shuffled by 1 kb block and Lowess-smoothed with a window size of 200 kb as a null distribution (black). The smoothed d scores (y-axis) were plotted along chromosome positions (x-axis).

We first examined whether constitutive CG methylation showed preference for certain chromosomal regions. The percent CG methylation for each of 1 Mb chromosome bins was calculated. Consistent with a previous report [15], methylation was generally high around pericentromeric regions and decreased toward chromosome arms (Figure 2A). The sharp decrease of methylation frequency immediately adjacent to pericentromere of chromosome 1 was probably due to high proportion of CNG methylation within this bin which was undetectable by our method (Figure 2A). For both SFPs and constitutive CG methylation, the trend of decreasing frequency from pericentromere toward euchromatin arms suggests potential purifying selection [1],[15]. Mutations within gene-rich regions are more likely to be deleterious, and based on studies in mammals cytosine methylated positions have a greater mutation rate [1]. In contrast to constitutive methylation, methylation polymorphisms exhibited little variation along chromosomes (Figure 2A). As DNA methylation could affect chromatin structure, such effect likely depends on dense methylation over long distance. To assess whether constitutive methylation sites exhibit co-methylation, i.e. broad regions with consistently methylated or unmethylated sites, we examined the distribution of enzyme effect d scores (modified t-statistics of enzyme effect) along chromosome positions by Lowess smoothing. Lowess smoothing performs locally weighted regression on neighboring d scores within an analyzed window (here 200 kb) so that each smoothed d score reflects the overall pattern of its neighbors. The smoothed enzyme effect d scores indicates significant regional methylation around pericentromeres, compared with a null distribution of smoothed d scores from random shuffling by 1 kb block (Figure 2B). Within euchromatin regions, however, the real distribution was indistinguishable from null distribution, indicating the lack of regional methylation (Figure 2B). We then evaluated the regional correlation of CG methylation polymorphism. In this context one accession may have increased or decreased regional methylation signal relative to the other strain. Lowess-smoothing of d scores for genotype×enzyme interaction effect revealed very few regional effects of CG methylation polymorphism (Figure S5A), suggesting that between genotypes methylation varies for individual loci rather than for large chromosome blocks. This result may be unique to our enzyme methylome approach which interrogates specific sites rather than anti-5methyl cytosine ChIP-chip which profiles methylation abundance within a ∼1 kb region.

**Figure 3:**
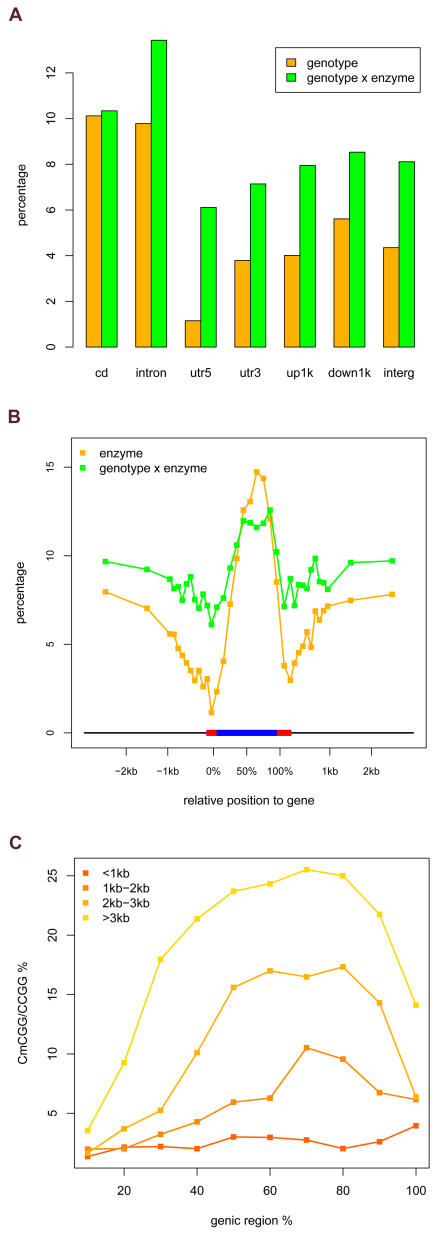
Genic Distribution of Constitutive and Polymorphic CG Methylation Sites. (A) Percent constitutive (orange) and polymorphic (green) CG methylation sites was calculated for seven annotation categories: coding sequence (CD); intron, 5′ UTR (utr5), 3′ UTR (utr3), sequence from transcriptional start to upstream 1 kb (up1k), sequence from transcriptional stop to downstream 1 kb (down1k), and inter-genic sequence (interg). (B) Percent constitutive (orange) and polymorphic (green) CG methylation sites was calculated along a typical gene. The results were based on all annotated genes possessing CCGG-containing feature(s) within the analyzed region. Gene sequences flanked by UTRs were divided to 10 percentiles based on position, upstream and downstream sequences were each divided to ten 100 bp intervals and two 1 kb intervals. Percent constitutive and polymorphic CG methylation sites was calculated for each interval and plotted as y-axis along a virtual gene of size 1900 bp (including 100 bp 5′UTR and 200 bp 3′UTR). Black lines: upstream and downstream sequences; red bar: UTRs; blue bar: gene sequences flanked by UTRs. The x-axis indicated the relative positions to the virtual gene. (C) Correlation between the size of gene (sequence flanked by UTRs) and the level of constitutive CG methylation. Genes possessing CCGG-containing feature(s) were separated to 4 groups based on their size. Within each group, gene regions were divided to 10 percentiles based on position, and the percent constitutive CG methylation (y-axis) was calculated for each percentile (x-axis).

We then examined whether methylation sites preferentially accumulated in specific genic intervals of the genome. Features were categorized based on genome annotation (coding sequence, intron, 5′ and 3′ UTR, and inter-genic regions). The percentage of features with constitutive CG methylation was calculated for each class. The extent of CG methylation varied among these categories: highest in coding sequences and introns, moderate in upstream (1 kb from transcriptional start site), downstream (1 kb from transcriptional stop site) and inter-genic regions, and very low in UTRs, especially 5′ UTR (Figure 3A, Table S4). Since coding sequences and introns are similar in CG methylation content, we refer to coding sequences and introns as genic regions in the following analysis. To examine the distribution of CG methylation in finer scale, genic regions were binned into ten percentiles based on relative position within the gene, and upstream and downstream sequences were each binned to ten 100 bp intervals and two 1 kb intervals. Percent CG methylation was calculated for each of these intervals. Methylation was extremely low in 5′UTRs and increased gradually until reaching a maximum near the third quarter of genes, and decreased sharply toward 3′UTRs (Figure 3B). Upstream and downstream regions beyond 1 kb showed moderate CG methylation (Figure 3B). Distribution of methylation polymorphisms among annotated sequence categories exhibited a similar pattern to that of constitutive methylation, except that introns seen to contain more polymorphic sites than exons (Figure 3A, Table S4). Along a typical gene, polymorphic methylation around gene ends was notably higher than constitutive methylation (Figure 3B), implying a potential role of methylation polymorphisms within these regions in regulating gene activity. To examine possible correlation between genic CG methylation and gene size [15], genes with CCGG-containing feature(s) were separated to 4 groups based on gene size. For each gene size group, genic regions were binned to 10 percentiles based on relative position, and the percent CG methylation for each bin was calculated. For genes smaller than 1 kb, methylation was low across the whole gene (Figure 3C). Methylation level generally increased with gene size, especially for the 3′ region of gene, while methylation within the 5′ region of gene adjacent to 5′ UTR maintained at low level (Figure 3C). Similar to constitutive methylation, methylation polymorphism generally increased with gene size (Figure S5B).

### Inheritance of CG Methylation Polymorphisms is Predominantly Additive

Considering the large number of polymorphic CG methylation sites within the genome, it is of interest to know how these polymorphic sites are inherited in the next generation. Dominant inheritance indicates that hybrids are more similar to one of the parents, while additive inheritance indicates that hybrids have intermediate phenotypes of parents. *Trans* methylation effects, perhaps due to differential activity of a cytosine-DNA-methyltransferase between accessions, might result in dominant methylation signatures in the F1 hybrids. Alternatively, *cis* methylation effects are more likely to be additive in hybrids, affecting a single inherited chromosome at the particular site. In Arabidopsis and likely other flowering plants, MET1-dependent maintenance of CG methylation is thought to be a default pathway, while activation of silenced genes within endosperm by specific demethylation of maternal allele has been observed for *MEA* and *FWA* [26],[27]. To examine these globally, we generated reciprocal F1 hybrids between Col and Van. F1 seedlings were grown together with parental strains, each cross direction with four maternal seed batch replicates. For each CCGG-containing feature, we modeled hybridization intensity by genotype, enzyme and genotype×enzyme interaction effects, where genotype effect was comprised of additive (contrasting parental strains), dominant (contrasting parental strains and F1 hybrids) and maternal (contrasting reciprocal F1 hybrids) effects. We named the difference between reciprocal F1s as maternal effect, merely because that maternal genotype is expected to have large influence in early development [28]. In the full model, the additive main effect detects differential signals between parental genotypes across enzyme treatments; thus detects SFPs. The enzyme main effect with greater *Hpa*II signal detects constitutive CG methylation, while differential CG methylation between contrasting groups is detected by corresponding interaction terms (explained in Figure S6). The additive×enzyme interaction again describes methylation polymorphisms between parental strains. With the inclusion of hybrid genotypes, we are particularly interested in the differential methylation between hybrids and parental lines (dominant×enzyme) and between reciprocal hybrid lines (maternal×enzyme). These terms reveal hybrid dominance methylation (Col or Van specific) or maternal specific methylation (Col or Van specific).

Although the additive by enzyme interaction again identified many significant methylation polymorphisms, for dominant by enzyme and maternal by enzyme interaction overall there was little evidence for an enrichment of significant scores for single loci compared with that expected by chance (Figure S7), indicating that inheritance of CG methylation is predominantly additive and has little or no maternal influence. Nonetheless, certain functional categories were enriched suggesting subtle dominance and maternal effects of methylation may exist (see below). We independently evaluated the CG methylation for F1 hybrids by PCR. F1 seedlings were grown with two maternal seed batch replicates for each reciprocal cross. Genomic PCR was performed using these F1 DNA samples after restriction enzyme digest. Although less quantitative, for the majority of 41 loci with confirmed CG methylation polymorphisms, methylation levels in F1 hybrids was intermediate to that of parental genotypes (Figure S2A and S2B). This is in agreement with our conclusion based on the modeling of array intensity that additive inheritance was predominant for polymorphic loci. In addition, methylation difference between reciprocal hybrids for the majority of these 41 loci was indistinguishable using our genomic PCR condition (Figure S2A and S2B). It should be noted, however, that in our experiment the plants used in the crosses to generate the parental lines and reciprocal F1 hybrid lines had been grown under a well controlled environment, and these plants were at about the same developmental stage at the time of cross (Materials and Methods). Environmental and developmental perturbation could potentially affect the variation and inheritance of methylome profile [19],[20].

### Correlation between CG Methylation and Gene Expression

**Figure 4:**
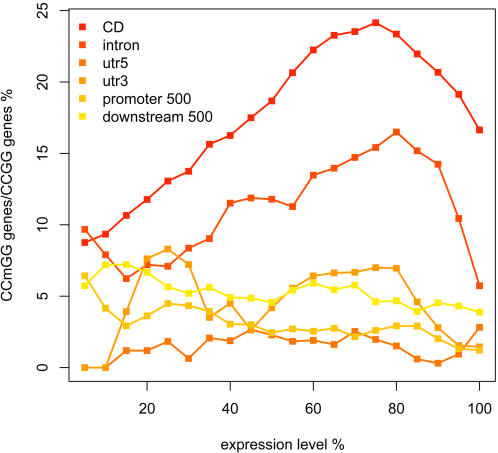
Correlation between Constitutive CG Methylation and Absolute Gene Expression Level. Genes possessing CCGG-containing feature(s) within analyzed sequence category were divided to 20 percentiles based on absolute gene expression level. Within each expression percentile (x-axis), percent genes with constitutive CG methylation site(s) was plotted as y-axis. The analyzed annotation categories were: coding sequences (CD); 5′ UTRs (utr5); 3′ UTRs (utr3); sequences from transcriptional start to upstream 500 bp (promoter 500); sequences from transcriptional stop to downstream 500 bp (downstream 500).

In the microarray experiment, the same seedling samples were split for enzyme methylome analysis and for expression profiling on the same microarray platform, allowing a direct comparison. We first examined the correlation between constitutive methylation and absolute gene expression level. Genes were divided into 20 percentiles according to their absolute expression levels. Within each expression percentile, the number of genes containing constitutive methylation site(s) within an analyzed annotation category was divided by the number of genes containing CCGG feature(s) within that category. For coding sequences and introns, absolute gene expression level was clearly correlated with degree of constitutive methylation (Figure 4): weakly expressed genes were the least methylated; methylation gradually increased with expression level then dropped sharply for highly expressed genes. Methylation within upstream/downstream sequences and UTRs was generally low across all expression percentiles (Figure 4). For the analyzed annotation categories, only UTRs in some cases had a small number of genes with CCGG-containing feature(s) in an expression percentile (Table S5), thus the result was unlikely affected by stochastic error in sampling.

**Figure 5:**
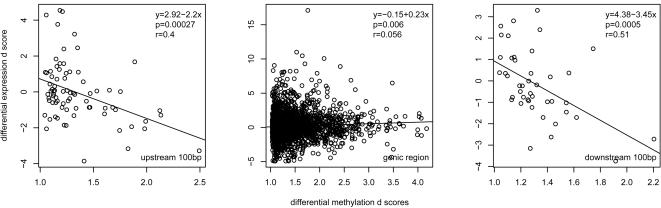
Correlation between CG Methylation Polymorphisms and Differential Gene Expressions. Differential gene expression d scores (y-axis) were linear regressed against d scores of genotype×enzyme effect (x-axis) for features within 100 bp upstream from transcriptional start (left panel), genic region (middle panel) and 100 bp downstream from transcriptional stop (right panel).

We further examined the correlation between methylation variation and expression variation. As the features significant for Van-specific methylation potentially represent duplicated regions within Van genome, we only focused on features significant (p<0.05) for Col-specific methylation. Differential gene expression d scores (modified t-statistics of differential gene expression between Col and Van) were linearly-regressed against genotype×enzyme interaction d scores. Analysis was performed separately for each of 100 bp intervals within upstream (Figure S8A) and downstream (Figure S8B) sequences and for genic regions. Significant negative correlation was observed for the 100 bp interval immediately upstream (r = 0.40, p = 0.00027; Figure 5 left panel), and for the 100 bp interval immediately downstream (r = 0.51, p = 0.00050; Figure 5 right panel). Methylation variation within genic regions showed a very weak, but significant, positive correlation with expression variation (r = 0.056, p = 0.0060; Figure 5 middle panel).

### Gene Set Enrichment

For a single gene, subtle difference in methylation or expression level between genotypes may not be detectable given the vast number of statistic tests. However, a coordinately regulated gene group may show a significant difference at the level of functional category. Parametric Analysis of Gene set Enrichment [29],[30] tests groups of genes that may individually exhibit small variation in the same direction and thus be biologically relevant. We applied PAGE to examine selective enrichment in gene ontology categories for constitutive CG methylation (Table S6A) and for additive, dominance, and maternal effects of CG methylation polymorphism (Table S6B). As the number of genes containing CCGG feature(s) within promoter (transcriptional start to 500 bp upstream) was relatively small for PAGE analysis (3,206 genes for biological process and 3,352 for molecular function), we focused on genes containing CCGG feature(s) within genic region (13,080 genes for 163 biological processes and 13,403 for 119 molecular functions). Genes with constitutive CG methylation was significantly enriched in binding activity such as nucleic acid binding, RNA binding and zinc ion binding , motor activity, aminoacyl-tRNA ligase activity, signal transducer activity, and ATPase activity (Table S6A). Comparison of gene set enrichment for additive, dominant and maternal effects of polymorphic CG methylation did reveal a few biological processes exhibiting dominant or parental-origin inheritance (Table S6B). For example, genes in flower development regulation exhibited greater CG methylation in Col parent, but greater CG methylation in Van-mother F1 hybrids (Table S7A). Genes in both cell redox homeostasis and ribosome biogenesis/assembly showed greater CG methylation in Col. In the F1 hybrids however CG methylation of cell redox homeostasis loci was close to Col parent while that of ribosome biogenesis/assembly loci was close to Van parent (Table S7A).

Gene set enrichment for gene expression polymorphisms also revealed specific functional categories as coordinately up or down regulated between genotype groups (Table S6C). Interestingly the categories identified included several that were also identified as enriched for methylation polymorphisms between the same genotype groups. For example, heat shock protein binding and microtubule motor activity were significant (p<1.67E-03 and p<7.56E-03, respectively) molecular functions with greater CG methylation in Col, which were also significant (p<5.84E-6 and p<9.64E-04, respectively) molecular functions with greater expression level in Col. Chlorophyll biosynthetic process and response to heat were significant (p<1.22E-03 and p<1.76E-02, respectively) biological processes with greater methylation in Col-mother F1, as well as significant (p<3.86E-06 and p<4.49E-09, respectively) biological processes with greater expression level in Col-mother F1. We did not observe overlap of enriched gene sets between dominance methylation and dominance gene expression. It is likely that for dominance expression, the effect of CG methylome was overly masked by large genetic regulatory effect. Furthermore, we observed many enriched functional categories for differential methylation between mothers that overlapped with enriched functional categories for differential expression between corresponding F1 hybrids (Table S7B). Thus, although individual sites showing genic CG methylation polymorphism had a subtle effect on gene expression, an underlying gene set coordination may exist dually affecting gene set expression and methylation profiles.

## Discussion

The fact that DNA methylation induces chromatin remodeling [5] implies potential DNA co-methylation over long distance. We observed high level of constitutive methylation blocks indicative of co-methylation around pericentromeric regions where transposons and repetitive elements accumulate. In addition, methylation polymorphisms within these regions were relatively low, indicating that constitutive dense methylation blocks might play an indispensable role in suppression of transposon activity. In contrast, euchromatin regions did not exhibit distinguishable blocks of co- methylation or co-regulated methylation polymorphisms, suggesting that effect of methylation within euchromatin regions might be locus-specific. Consistent with our results, methylome profiling in human tissues and cell lines also demonstrated the lack of co-methylation beyond 1 kb distance [18]. Nevertheless, co-regulation of DNA methylation over long distance in euchromatin regions was suggested by Regions of IncreaseD Gene Expression (RIDGEs) where physical gene clusters are expressed at high level [31],[32]. Such epigenetic regulation of large chromosome blocks, however, could depend on spatial or temporal signals [33], or depend on epigenetic mechanisms other than CG methylation.

A recent study by Vaughn et al. did not observe a relationship between differential DNA methylation in euchromatin regions and differential gene expression [21]. In contrast, we found that there is a significant negative correlation between the degree of methylation variation within immediate upstream/downstream regions and the degree of expression variation. The earlier study was based on the analysis of expression data for biological samples grown in different experiment and was limited to 317 genes on chromosome 4. In this study, we evaluated the correlation between methylation variation and expression variation using a quantitative comparison of expression and methylation profiles for ∼4,000 genes and for more than 400 promoters which contained polymorphic CG methylation site(s). In addition, our expression and methylation data were obtained from matched samples grown in the same experiment, eliminating the confounding effects of development stage and environmental condition. Methylation within immediate upstream/downstream regions could interfere with the transcription initiation/termination, which was suggested by our observation of low constitutive methylation levels within these regions. The negative correlation between expression variation and methylation polymorphism within upstream/downstream regions indicates that CG methylation polymorphisms within these regions could play a role in regulating gene expression. Direct repression of basal transcription by DNA methylation within immediate upstream region was also supported by biochemical studies [6]. Methyl-binding proteins could exert a large effect inhibiting gene expression, as seen in human cells for example [7], but efficient binding of these mediators to methylated promoters may require many methylated sites. Finally, methylation effects of gene expression may not be immediate. Developmentally and/or environmentally induced physiological signals may separate a coordinated response.

CG methylation within genic regions is notably high, and exhibits a clear trend, increasing from 5′ to 3′ in longer genes. The exact biological function of genic CG methylation, however, remains elusive. Several biochemical studies demonstrated that intra-genic methylation decreases the efficiency of transcription elongation [10],[11],[34]. Nevertheless, in all these studies the examined sequences were methylated at most of their cytosine residues and such dense methylation induced closed chromatin structure [11]. In contrast, genic CG methylation in Arabidopsis occurs at discrete CG clusters [21],[25], which has been proposed to prevent transcription from cryptic promoters [15],[25]. Under this model, weakly expressed genes as well as highly expressed genes are less methylated; the formation of transcriptional initiation complex on cryptic promoters is constrained either by a closed chromatin structure, or by densely occupied DNA strands containing the transcription elongation machinery [15]. In our study, several lines of evidence also implied that genic CG methylation is consistent with increased transcription processability: genic CG methylation increased with gene size and primarily occurred at the 3′ of gene; except for highly expressed genes, correlation between absolute expression level and constitutive CG methylation was positive; although for individual genes positive correlation of expression variation and CG methylation variation was very weak, such correlation was frequently seen at the level of functional categories. The positive effect of genic CG methylation on gene expression, however, is compensated by the fact that dense methylation eventually induces a more closed chromatin structure to impede transcription elongation. It is possible that these two effects jointly decide the efficiency of transcriptional elongation.

Gene expression regulatory networks are comprised of *cis-* and *trans-*acting factors which exert immediate and large effects on gene expression. Such regulatory networks, however, are exposed to fluctuations stemming from internal and external signals. In contrast, DNA methylation is thought to be relatively stable. Although here we find the direct effect of DNA methylation on expression is subtle, its effect may persist through development directly or indirectly regulating expression and altering whole plant phenotypes. A clear example is epigenetic control of *FLC* expression which affects flowering time [35],[36]. In the other hand, life history and environment could accumulatively alter DNA methylation profile [37]. Thus, CG methylation could serves as a memory mechanism in the genome to propagate developmental and environmental influences by modulating gene expression plasticity. The co-enriched functional categories for expression variation and for genic CG methylation polymorphisms further suggest the possible contribution of DNA methylation polymorphisms to natural gene expression variation.

Recent epigenetic studies in Arabidopsis have made significant contribution in revealing genome-wide DNA methylation patterns. Nevertheless, more large scale genomic and genetic experiments are essential to understand the dynamics and biological functions of DNA methylome. Particularly, it is of great interest to understand how epigenetic regulation of gene activity directly controls or is affected by developmental programs and environmental responses. Finally the genetic architecture underlying natural variation of DNA methylation is unknown. Our approach for simultaneous profiling of genetic, epigenetic, and transcriptional polymorphisms provides an initial effort toward such an understanding by leveraging a powerful microarray platform.

## Materials and Methods

### Plant Materials

Seeds of *Arabidopsis thaliana* accessions Col-0 (accession number CS22625) and Van-0 (accession number CS22627) were obtained from Arabidopsis Biological Resource Center. Seeds were planted in soil, imbibed for 5 days in cold room at 4°, and moved to green house in January 31, 2005. Plants were grown in green house with 16 h light (cool white light supplemented with incandescent) and 8 h dark at constant temperature of 20°. The first cross experiment was conducted in February 28, 2005, and in March 1, 2005 the second cross experiment was conducted between the same plant pairs as in the first experiment. Both cross experiments began around 9:00am and ended around 5:00pm. In each cross experiment, four replicate crosses for each of Col×Col, Van x Van, Van (♀)×Col (♂), and Col (♀)×Van (♂) were made. Each replicate cross was between individual paternal and maternal plant and each parental plant was only used once (16 Col and 16 Van plants used in total). For each replicate cross, the seeds from the two experiments were combined and used as one maternal seed batch.

∼250 seeds from each maternal seed batch were grown on a single petri dish. After gas sterilization for 4 h seeds were plated on a total of 16, 0.7% agar (Sigma) plates supplemented with 0.5 X Murashige and Skoog salts (Sigma). Seed plates were placed horizontally in a growth chamber (Percival Scientific Inc., model E361) after stratification for 5 days at 4°. Seedlings were grown for 78 hours under a diurnal mode with 12 h light (cool white light supplemental with red light) and 12 h dark at a constant temperature of 20°.

### Sample Preparation and Microarray Hybridization

Seedlings grown on each plate were split for genomic DNA and RNA preparation. ∼100 seedlings from each plate were pooled and genomic DNA was extracted using DNeasy plant mini kit (Qiagen). About 300 ng DNA was digested with 10 units of *Hpa*II or *Msp*I (New England Biolabs) in 50 uL volume at 37° for 16 h. Restriction enzymes were inactivated by heating at 65° for 20 min. DNA was ethanol-precipitated and rinsed with 80% ethanol. DNA was dissolved in 72 uL distilled water and subjected to labeling using BioPrime DNA labeling system (Invitrogen) with conditions modified as previously described [38]. About 20 ug total RNA was isolated from an additional 120 seedlings per plate using RNeasy plant mini kit (Qiagen). Poly-(A) RNA was enriched from total RNA using Oligotex mRNA mini kit (Qiagen). Poly-(A) RNA was mixed with 166 ng random hexamer (Invitrogen) and subjected to first-strand cDNA synthesis (Invitrogen) as manufacturer recommended in a total volume of 40 uL at 42° for 1 h. The 40 uL first-strand reaction was used in second-strand cDNA synthesis (Invitrogen) as manufacturer recommended in a total volume of 300 uL at 16° for 2 h. Samples were then subjected to RNase treatment at 37° for 20 min with 20 units RNaseH (Epicentre), 1 unit RNaseA and 40 units RNaseT (Ambion). Double-stranded cDNA was further purified using Qiaquick PCR purification kit (Qiagen), and then labeled using BioPrime DNA labeling system (Invitrogen) as described above. About 30 ug labeling product from enzyme-treated genomic DNA or from double-stranded cDNA was subjected to hybridization to Arabidopsis Tiling 1.0F array (Affymetrix) using standard gene expression array washing/staining protocol (Affymetrix). Thus we used a total of 32 chips for genomic DNA sample hybridization and an additional 16 chips for RNA sample hybridization.

### Genomic PCR, Epityper, and Bisulfite Sequencing

Seeds from the same maternal seed batches used in the microarray experiments were gas sterilized, plated and stratified as described above. Seedlings were grown in the same growth chamber for 78 h under the same condition settings as in microarray experiments. About 100 seedlings from each plate were pooled, froze in liquid nitrogen and stored at -80° till genomic DNA preparation. This growth and harvest procedure was repeated in a separate experiment. For each sample from each growth experiment, genomic DNA was extracted. Genomic DNA samples from one growth experiment were used for genomic PCRs and bisulfite sequencing. For genomic PCR, ∼300 ng DNA sample was digested by *Hpa*II and *Msp*I as described above. 0.1 uL digestion reaction or 0.1 uL mock digestion reaction without restriction enzyme was used as template in PCR, with 0.1 uL extaq (Takoma, Japan) in 10 uL volume. PCR condition was set for denature 94° 3 min, 28 cycles of: 94° 15 s, 62° 15 s, 72° 20 s, extend 72° 5 min. 2 uL PCR reaction was separated on 1.2% agrose gel (Invitrogen). For bisulfite sequencing, ∼100 ng genomic DNA was converted using EZ DNA Methylation Gold Kit (Zymo Research). Strand-specific PCR was performed as previously described [39]. PCR products were gel purified and cloned using TOPO kit (Invitrogen), and 10-15 clones per template were sequenced. Genomic DNA samples from both growth experiments were submitted to Sequenom for epityper analysis (http://www.sequenom.com/Seq_methylation.html).

### Microarray Data Analysis

The microarray data analysis described below used R scripts (Text S1; also available online http://naturalvariation.org/ccggMethylome).

Perfect match probes from Arabidopsis tiling 1.0F array (Affymetrix) were megablasted against Arabidopsis genome release version 7 including mitochondria and chloroplast sequences with word size > =  8 and E-value < =  0.01. Single perfect matches, without a 2nd partial match of>18/25 bp were selected giving a total of 1,683,620 unique probes. These were mapped to annotated mRNAs as intron, transcription unit (exon, alternative exons), inter-genic region, or flanking probes which span an annotated boundary. Only transcription unit probes were used for expression analysis.

For each chip used for genomic hybridization, the CEL intensity of 1,683,620 unique probes was corrected to remove background effects [22]. Intensity across 32 chips (4 genotypes×4 replicates×2 enzymes) was then normalized by quantile normalization using Bioconductor package Affy. For 1,683,620 probes, SFPs were detected using Bioconductor package Siggenes [40]. A total of 54,519 unique probes contain CCGG within their sequence. For detection of constitutive and polymorphic CG methylation between Col and Van, intensity for each CCGG probe was fit by a mixed linear effect model of genotype+enzyme+genotype×enzyme+random effect (plant). The genotype effect contrasts two lines, and enzyme effect contrasts two enzyme treatments. For each fixed effect, a modified t statistic was calculated for each probe as d = effect coefficient /(standard deviation+s0), where s0 was a small constant set as the 5% quantile of standard deviations across 54,519 CCGG probes and 1000 permutations (see below) . The adding of s0 in the denominator makes sure that probes with very small observed errors are not called significant [41]. To evaluate the statistic significance of the d scores for an effect, we calculated a nominal p value based on permutation, where for each probe the p value of the effect was defined as the proportion of d scores, across all CCGG probes and all permutations, which were more extreme than the real d score. For permutation the plant random effect was removed first, then the procedure involved: 1) fitting a partial model missing the effect being tested; 2) permuting residuals; 3) adding permutated residuals to the predicted values; 4) fitting that data with a full model; 5) calculating a d score; 6) repeating step 2 to 5 for 1,000 times. The null hypothesis here is that the effect being tested is not significant, thus residuals from partial modeling are assumed to be independent random variables that could be permutated across samples.

For analysis of inheritance of CG methylation polymorphisms, intensity for each CCGG probe was linear regressed by the same mixed linear effect model of genotype+enzyme+genotype×enzyme+random effect (plant), where genotype = additive+dominant+maternal. Additive effect contrasts between parental genotypes, dominant effect contrasts between average of parental genotypes and average of F1 reciprocal hybrids, maternal effect contrasts between F1 reciprocal hybrids. To evaluate the statistic significance for each effect, the same permutation approach described above was used.

For each chip from cDNA hybridization, CEL intensity of 1,683,620 unique probes was corrected to remove background effects as described above. SFPs detected from genomic DNA hybridization were removed from transcription unit probes. Intensity for remaining transcription unit probes was normalized across 16 chips by quantile normalization using Bioconductor package affy. For the annotated genes with more than 3 probes, probe intensity from each probe set was modeled by additive, dominant and maternal effect. For each gene, d score was calculated as described above, with s0 set to 50% of standard deviation over 1000 permutations.

For the genome and genic distribution of methylation, chromosome position of second cytosine of the first CCGG sub-sequence (only 1,010 probes contain > = 2 CCGG sub-sequence) of each probe was mapped to annotation categories based on information from TAIR blast_datasets of version 7. For correlation between absolute expression value and constitutive CG methylation, the expression value for each gene was the mean of exon probe intensity across the probe set and genotypes, and the percent CG methylation in Figure 4 was obtained by three point average for presentation purpose, which doesn't change the result. For correlation between CG methylation polymorphism and gene expression polymorphism, the differential methylation d score was averaged across a gene.

For parametric analysis of gene set enrichment, the d scores for effect under study were used as summary statistics.

### Accession Numbers

The Gene Expression Omnibus (GEO) (http://www.ncbi.nlm.nih.gov/geo) accession numbers discussed in this paper are GSE8890 and GSE8891.

## Supporting Information

Figure S1 Experimental Aspects Potentially Affect Methylation Detection. (A) The distribution of CCGG features on Arabidopsis 1.0F array. Each chromosome was binned to 100 kb bins. For each bin the percent CCGG-containing feature (y-axis) was plotted along chromosome positions (x-axis). The percent CCGG-containing features was defined as the number of CCGG-containing features within the bin divided by the total number of unique features within the bin. (B) Detection of constitutive and polymorphic CG methylation sites was not significantly affected by the fragment length variation caused by enzyme digestion. For each of analyzed CCGG sites, the distance between its two flanking CCGG sites was calculated (based on Col genomic sequences). The d scores (y-axis) for constitutive methylation (left panel) or for polymorphic methylation (right panel) were grouped by the distance of their flanking CCGG sites (x-axis). (C) Effect of relative CCGG position within features. The CCGG-containing features on Arabidopsis 1.0F array were grouped by the position of their second cytosine within the features. For each position group (x-axis), the number of features (left panel), the constitutive methylation d scores (middle panel) or the polymorphic methylation d scores (right panel) were plotted as y-axis.
(0.15 MB PDF)

Figure S2 Verification of Methylation Polymorphisms by Genomic PCR. Primers were designed to flank the selected CCGG-containing features, with only one CCGG sequence within a flanked region. F1v: F1 hybrids with Van as mother; F1c: F1 hybrids with Col as mother; control: genomic DNA without enzyme digestion; *Hpa*II: genomic DNA digested by *Hpa*II; *Msp*I: genomic DNA digested by *Msp*I. (A) Verification of Col-specific methylation. 24 loci were randomly selected from the features significant (p<0.03) for Col-specific methylation. (B) Verification of Van-specific methylation. 17 loci were randomly selected from the features significant (p<0.03) for Van-specific methylation. (C) Evaluation of false negative rate. 33 loci were randomly selected from all 54,519 CCGG-containing features.
(0.50 MB TIF)

Figure S3 Verification of Methylation or Methylation Polymorphisms by Quantitative Measurement. (A-C) Verification of methylation or methylation polymorphisms by epityper. The quantification scale of cytosine methylation was illustrated in the top panel. The CCGG site detected by microarray experiment was boxed. The corresponding gene and the relative position of the detected CCGG site within gene were illustrated in the bottom panel. For reciprocal hybrid lines, mother strain was listed first. Plant samples grown in two independent growth experiments were used in the epityper analysis. (A) polymorphic locus chr1/ 9491334; (B) polymorphic locus chr4/10420079; (C) constitutive locus chr1/22476369. (D-F) Verification of methylation polymorphisms by bisulfite sequencing. The percent methylation for all cytosine residues (y-axis) was plotted on their relative positions within the segment (x-axis). The CCGG site tested was pointed by the black arrow. (D) locus chr1/18407942; (E) locus chr1/27257856; (F) locus chr/30092640.
(0.34 MB PDF)

Figure S4 The Size Distribution of ChIP-Chip Segments [16] with Constitutive (Upper Left) or Polymorphic (Lower Left) CG Methylation Sites Detected by our Method. The y-axis indicated the number of segments; the x-axis indicated the ChIP-chip segment size. The size distribution of ChIP-chip segments which contained CCGG sites analyzed in our study but were without constitutive (upper right) or polymorphic (lower right) CG methylation sites detected by our method were also presented.
(0.39 MB TIF)

Figure S5 Distribution of Polymorphic CG Methylation Sites. (A) Co-regulation of methylation polymorphisms along chromosomes. The d scores of genotype × enzyme effect for 54,519 CCGG-containing features were Lowess-smoothed with a window size of 200 kb (green), or were shuffled by 1 kb block and Lowess-smoothed as a null distribution (black). The smoothed d scores (y-axis) were plotted along chromosome positions (x-axis). (B) Correlation between the size of gene (sequence flanked by UTRs) and the level of polymorphic CG methylation. Genes possessing CCGG-containing feature(s) were separated to 4 groups based on their size. Within each group, gene regions were divided to 10 percentiles based on position, and the percent polymorphic CG methylation (y-axis) was calculated for each percentile (x-axis).
(0.08 MB PDF)

Figure S6 Diagram of each Effect in the (Additive+Dominant+Maternal) × Enzyme Full Model. F1c: F1 hybrids with Col-mother; F1v: F1 hybrids with Van-mother. Additive effect indicates hybridization intensity difference between parents across enzyme treatments, suggesting SFP. Dominant effect indicates hybridization intensity difference between the mid-parent (dashed line) and the average of reciprocal F1 hybrids across enzyme treatments. Maternal effect indicates hybridization intensity difference between reciprocal F1 hybrids across enzyme treatments. Enzyme effect with greater *Hpa*II signal indicates constitutive CG methylation; that with greater *Msp*I signal was likely due to normalization. Additive × enzyme effect indicates differential CG methylation between parents; dominant × enzyme effect indicates differential CG methylation between mid-parents and average of F1 hybrids; maternal × enzyme effect indicates differential CG methylation between reciprocal F1 hybrids.
(0.10 MB PDF)

Figure S7 The Quantile-Quantile Plot for each Effect in the (Additive+Dominant+Maternal) × Enzyme Full Model. The x-axis represents the quantiles of null d scores obtained from 1000 permutation; y-axis represents the quantiles of real d scores for each of main effects and interaction effects.
(0.04 MB TIF)

Figure S8 Correlation of Methylation Variation and Expression Variation. Differential gene expression d scores (y-axis) were linear regressed against polymorphic CG methylation d scores (x-axis) for features within (A) 100 bp intervals starting from upstream 100 bp to upstream 1000 bp; (B) 100 bp intervals starting from downstream 100 bp to downstream 1000 bp.
(0.14 MB PDF)

Table S1 SFPs between Col and Van. (A) SFPs detected at different FDR. ^a^The number of features called significant. ^b^The number of features determined as false positives based on permutation. ^c^The number of features called significant with either greater Van signals (Sig-) or greater Col signals (Sig+). (B) Genic distribution of 120,810 SFPs detected at 1% FDR with greater Col signal. ^a^The number of SFPs within each annotation category. The annotation categories analyzed included coding sequences (CD), intron, UTRs, transcriptional start to upstream 1 kb (promoter), transcriptional stop to downstream 1 kb (downstream) and inter-genic regions. As genes on forward and reverse direction could overlap, the total number of SFP mapped to these annotation categories was more than 120,810. ^b^The number of features within each annotation category. The total number of features mapped to annotation categories was more than 1,683,620 due to overlap between two strands.
(0.02 MB XLS)

Table S2 The Counting Tables for Loci Validated by Epityper or Bisulfite Sequencing.
(0.05 MB XLS)

Table S3 Comparisons with Published ChIP-Chip Data. Set 1 was comparison with data by Zhang et al. [16]; set 2 was comparison with data by Zilberman et al. [15]. EE: enzyme effects, i.e. the constitutive CG methylation sites detected in this study. ^a^The p-value thresholds of enzyme effects. ^b^The number of enzyme effects detected at different p-value thresholds. ^c^The number of enzyme effects within the methylation regions detected by ChIP-chip methods. ^d^The number of CCGG-containing features within the methylation regions detected by ChIP-chip methods. ^e^The number of enzyme effects outside of the methylation regions detected by ChIP-chip methods. ^f^The number of CCGG-containing features outside of the methylation regions detected by ChIP-chip methods. ^g^The p value of the χ^2^ test for enrichment of enzyme effects in ChIP-chip regions. ^h^The coverage of CCGG sites within ChIP-chip regions by enzyme effects. ^i^The proportion of enzyme effects outside of ChIP-chip regions.
(0.02 MB XLS)

Table S4 Genic Distribution of Constitutive and Polymorphic CG Methylation Sites. The analyzed annotation categories included coding sequences (CD), intron, UTRs, transcriptional start to upstream 1 kb (promoter), transcriptional stop to downstream 1 kb (downstream) and inter-genic regions. ^a^The number of features significant (p<0.05) for constitutive CG methylation. ^b^The number of CCGG-containing features. ^c^The number of features significant (p<0.05) for polymorphic CG methylation.
(0.02 MB XLS)

Table S5 For Gene Expression Percentiles the Number of Genes with CCGG-Containing Feature(s) within the Analyzed Sequence Category. The analyzed annotation categories included coding sequences (CD), intron, UTRs, transcriptional start to upstream 500 bp (promoter), transcriptional stop to downstream 500 bp (downstream). ^a^The 20 percentiles based on absolute gene expression level.
(0.01 MB XLS)

Table S6 Gene Set Enrichment in GO Categories. (A) Gene set enrichment in GO categories for constitutive CG methylation within genic region. (B) Gene set enrichment in GO categories for polymorphic CG methylation within genic region. (C) Gene set enrichment in GO categories for gene expression variation.
(0.05 MB XLS)

Table S7 Overlap in Enriched Gene Sets. (A) Non-additive inheritance of CG methylation variation in gene sets. Biological processes significantly (p<0.05) enriched for differential CG methylation within genic regions. ^a^GO identification number. ^b^Biological processes with greater methylation in Col (Col>Van), or in Van (Van>Col). ^c^Biological processes with greater methylation in F1 hybrids (F1 hybrids >parents), or in parents (parents>F1 hybrids). ^d^Biological processes with greater methylation in Col-mother F1 (Col-mother F1>Van-mother F1), or in Van-mother F1 (Van-mother F1>Col-mother F1). (B) Contribution of mother methylome profile to offspring gene expression profile. The GO terms marked with asterisk were overlapped categories between differential methylation and differential expression. ^a^GO biological process categories. ^b^GO molecular function categories. ^c^The top 6 biological processes and top 6 molecular functions with greater CG methylation in Col than in Van. ^d^The top 6 biological processes and top 8 molecular functions with greater expression level in Col-mother F1 than in Van-mother F1. ^e^GO identification number.
(0.03 MB XLS)

Text S1 R Scripts for Microarray Data Analysis.
(0.11 MB TXT)
