# Correction: Global Analysis of Genetic, Epigenetic and Transcriptional Polymorphisms in *Arabidopsis thaliana* Using Whole Genome Tiling Arrays

**DOI:** 10.1371/annotation/e21d3565-fec6-44d9-8fab-83da49c7c0b8

**Published:** 2008-06-10

**Authors:** Xu Zhang, Shin-Han Shiu, Andrew Cal, Justin O. Borevitz

The second author's name was incorrect. It should be Shin-Han Shiu. The correct citation is shown here:

Zhang X, Shiu SH, Cal A, Borevitz JO (2008) Global Analysis of Genetic, Epigenetic and Transcriptional Polymorphisms in *Arabidopsis thaliana* Using Whole Genome Tiling Arrays. PLoS Genet 4(3): e1000032. doi:10.1371/journal.pgen.1000032

