# Supplementary figures and images for: Global Analysis of Genetic, Epigenetic and Transcriptional Polymorphisms in Arabidopsis thaliana Using Whole Genome Tiling Arrays
Source: PLoS Genet. 2008 Mar 21;4(3):e1000032. doi: 10.1371/journal.pgen.1000032 (PMC2265482; doi:10.1371/journal.pgen.1000032)

**A**

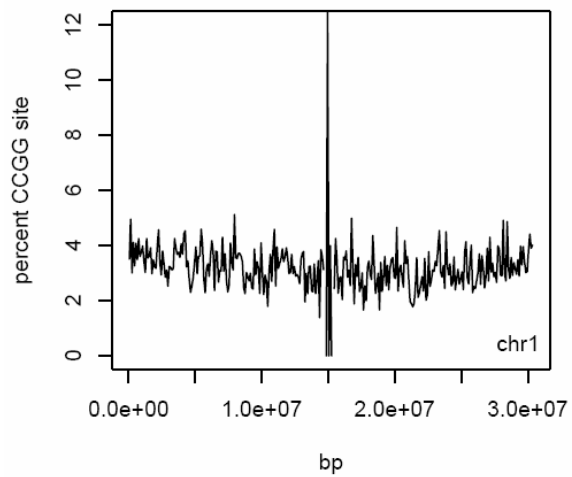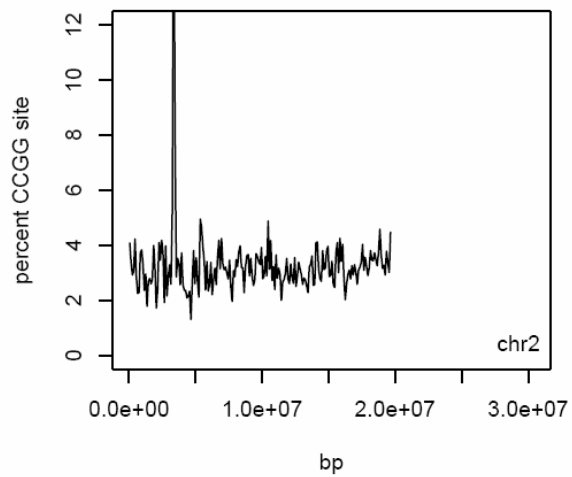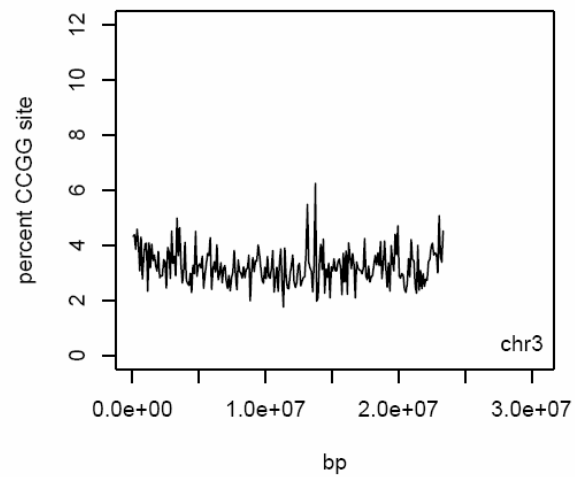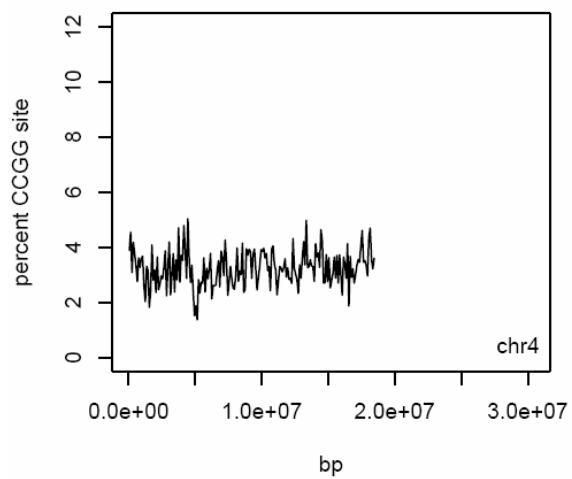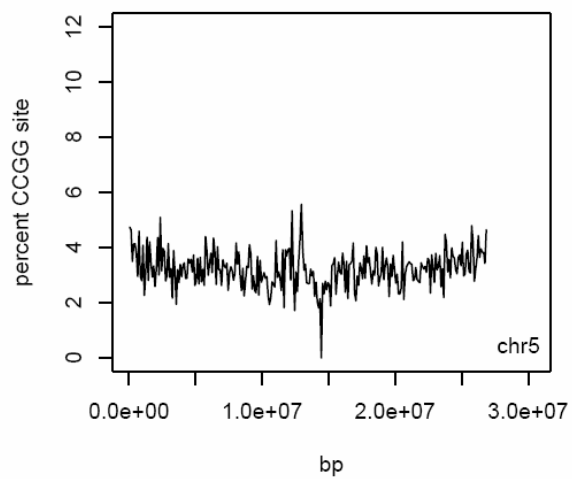

**B**

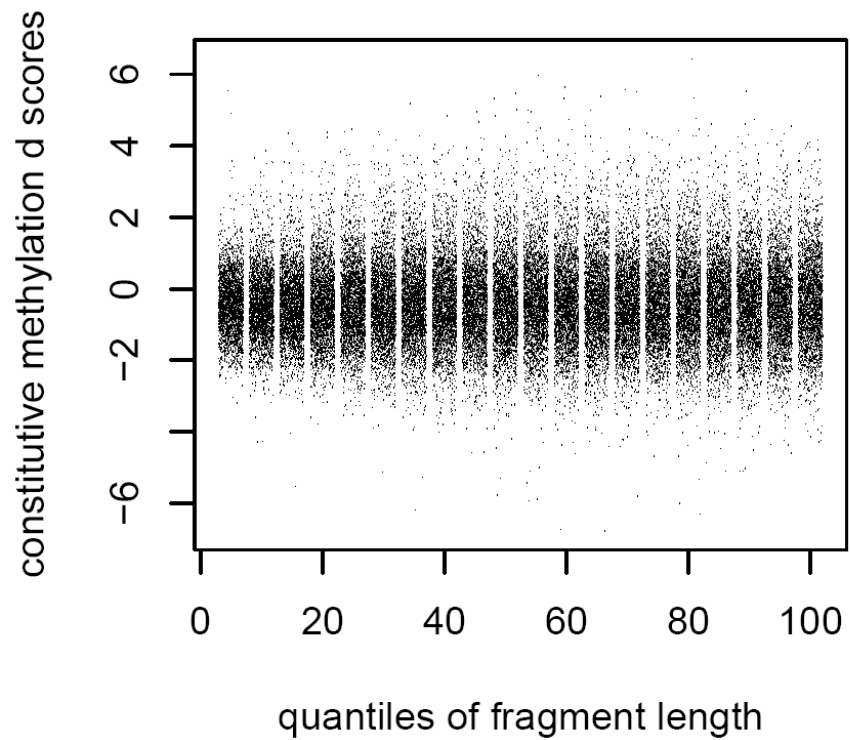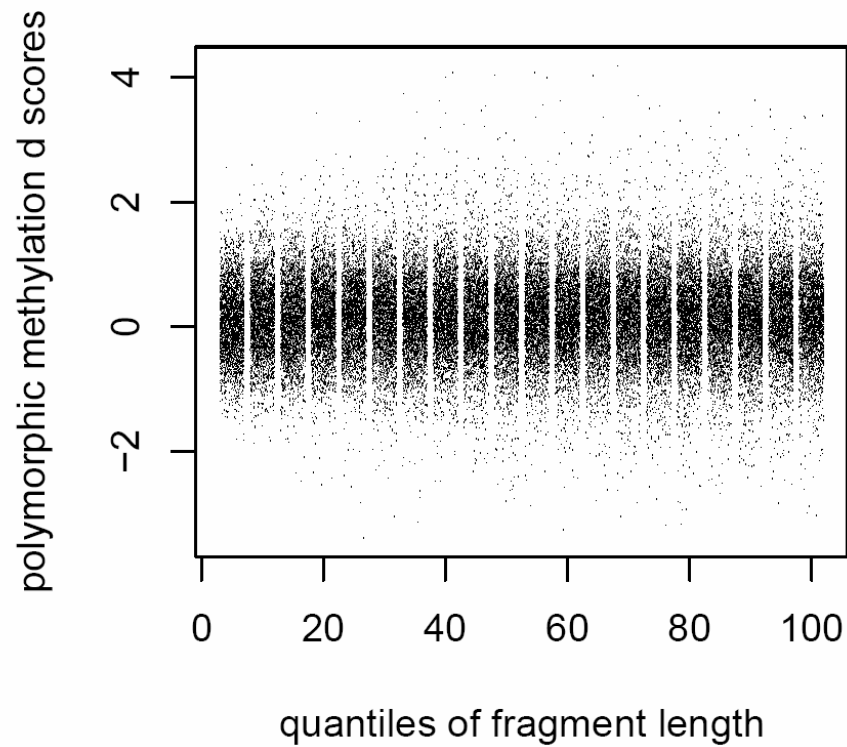

**c**

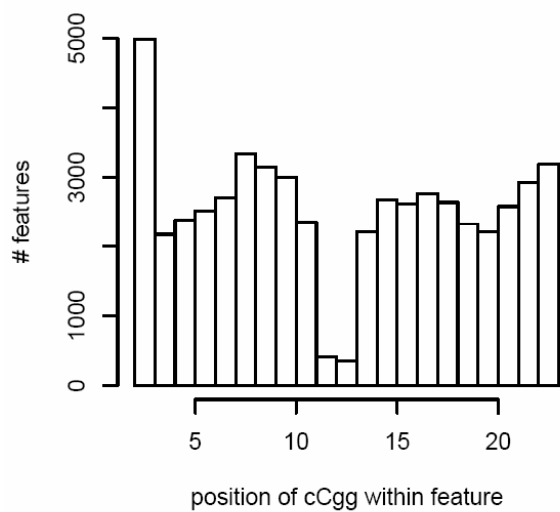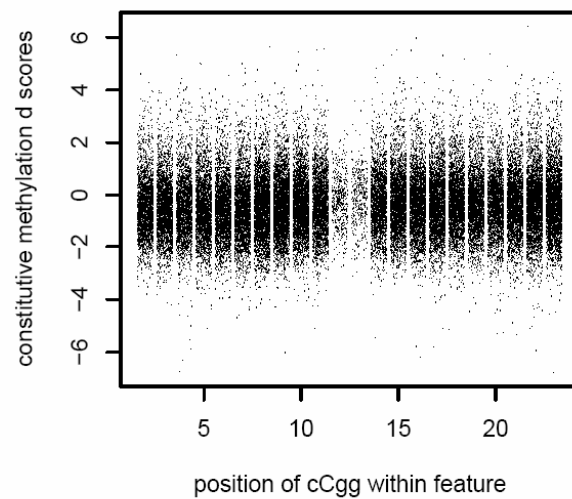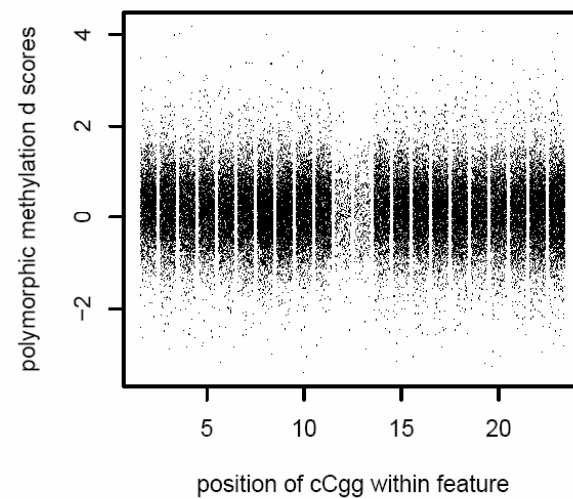

Supplement: Figure S1 — Experimental Aspects Potentially Affect Methylation Detection. (A) The distribution of CCGG features on Arabidopsis 1.0F array. Each chromosome was binned to 100 kb bins. For each bin the percent CCGG-containing feature (y-axis) was plotted along chromosome positions (x-axis). The percent CCGG-containing features was defined as the number of CCGG-containing features within the bin divided by the total number of unique features within the bin. (B) Detection of constitutive and polymorphic CG methylation sites was not significantly affected by the fragment length variation caused by enzyme digestion. For each of analyzed CCGG sites, the distance between its two flanking CCGG sites was calculated (based on Col genomic sequences). The d scores (y-axis) for constitutive methylation (left panel) or for polymorphic methylation (right panel) were grouped by the distance of their flanking CCGG sites (x-axis). (C) Effect of relative CCGG position within features. The CCGG-containing features on Arabidopsis 1.0F array were grouped by the position of their second cytosine within the features. For each position group (x-axis), the number of features (left panel), the constitutive methylation d scores (middle panel) or the polymorphic methylation d scores (right panel) were plotted as y-axis. (0.15 MB PDF) [file pgen.1000032.s001.pdf]

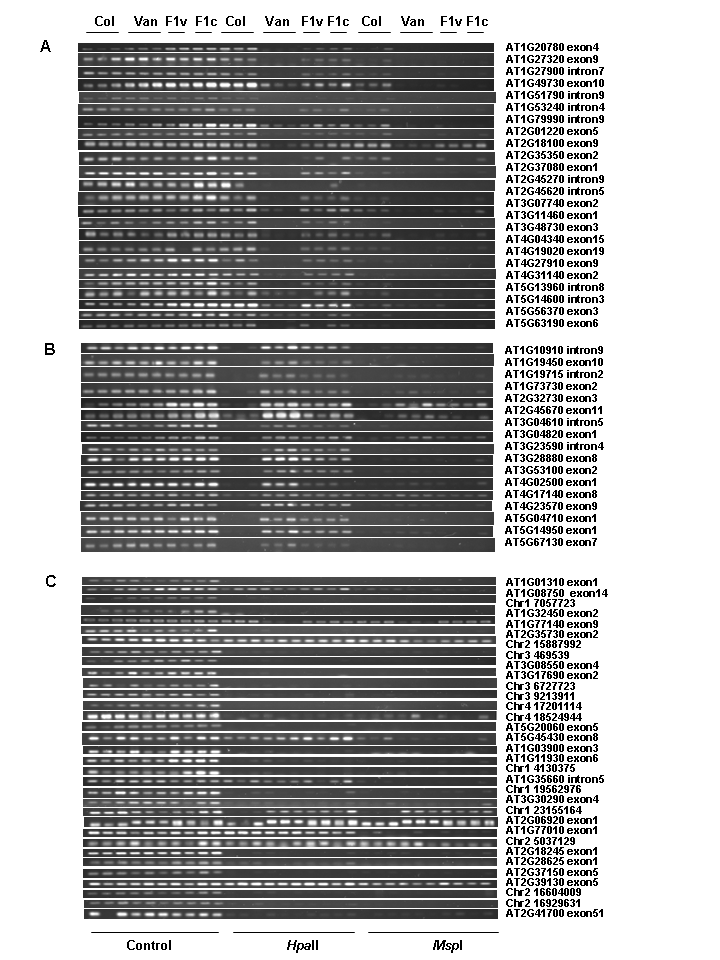

Supplement: Figure S2 — Verification of Methylation Polymorphisms by Genomic PCR. Primers were designed to flank the selected CCGG-containing features, with only one CCGG sequence within a flanked region. F1v: F1 hybrids with Van as mother; F1c: F1 hybrids with Col as mother; control: genomic DNA without enzyme digestion; HpaII: genomic DNA digested by HpaII; MspI: genomic DNA digested by MspI. (A) Verification of Col-specific methylation. 24 loci were randomly selected from the features significant (p<0.03) for Col-specific methylation. (B) Verification of Van-specific methylation. 17 loci were randomly selected from the features significant (p<0.03) for Van-specific methylation. (C) Evaluation of false negative rate. 33 loci were randomly selected from all 54,519 CCGG-containing features. (0.50 MB TIF) [file pgen.1000032.s002.tif]

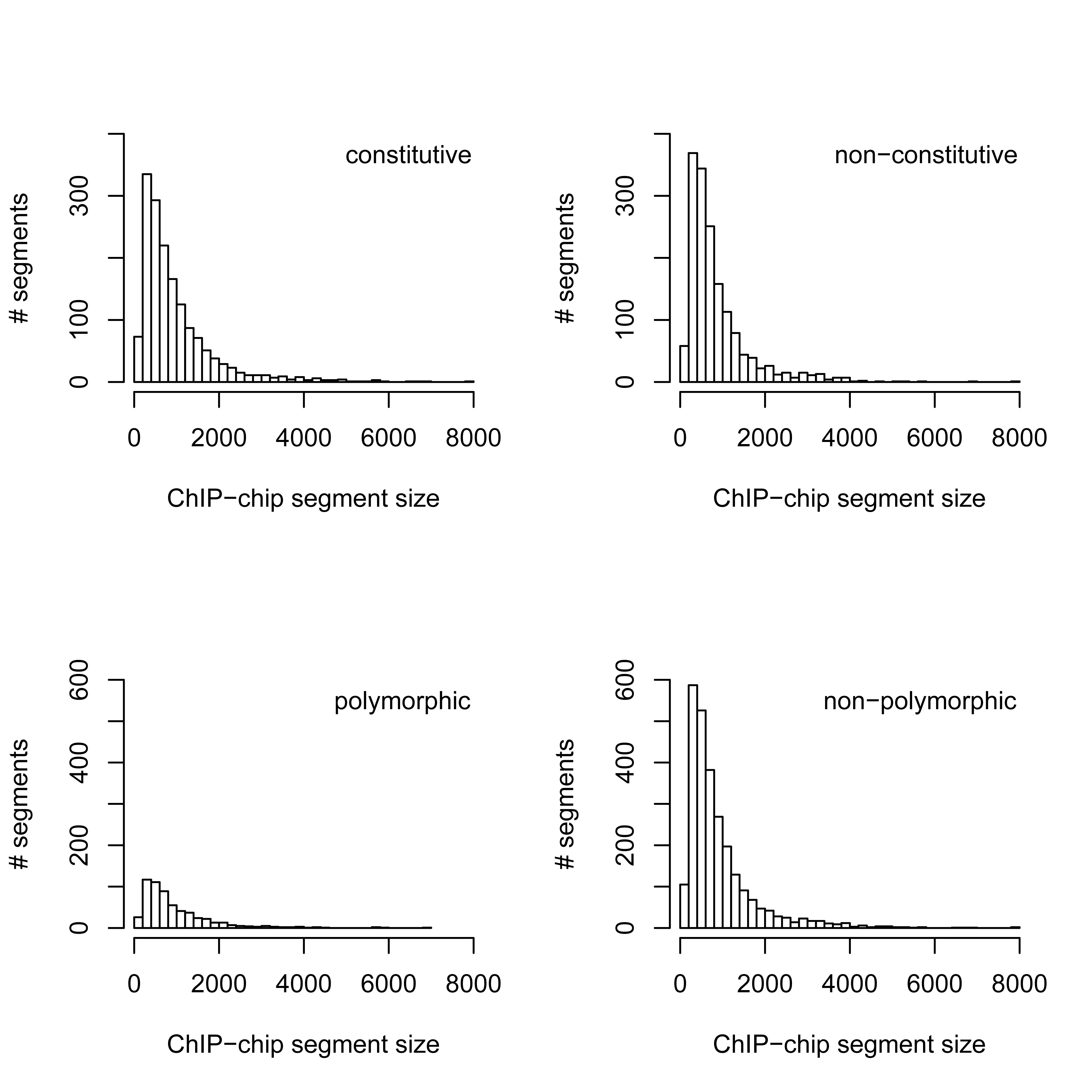

Supplement: Figure S4 — The Size Distribution of ChIP-Chip Segments [16] with Constitutive (Upper Left) or Polymorphic (Lower Left) CG Methylation Sites Detected by our Method. The y-axis indicated the number of segments; the x-axis indicated the ChIP-chip segment size. The size distribution of ChIP-chip segments which contained CCGG sites analyzed in our study but were without constitutive (upper right) or polymorphic (lower right) CG methylation sites detected by our method were also presented. (0.39 MB TIF) [file pgen.1000032.s004.tif]

A

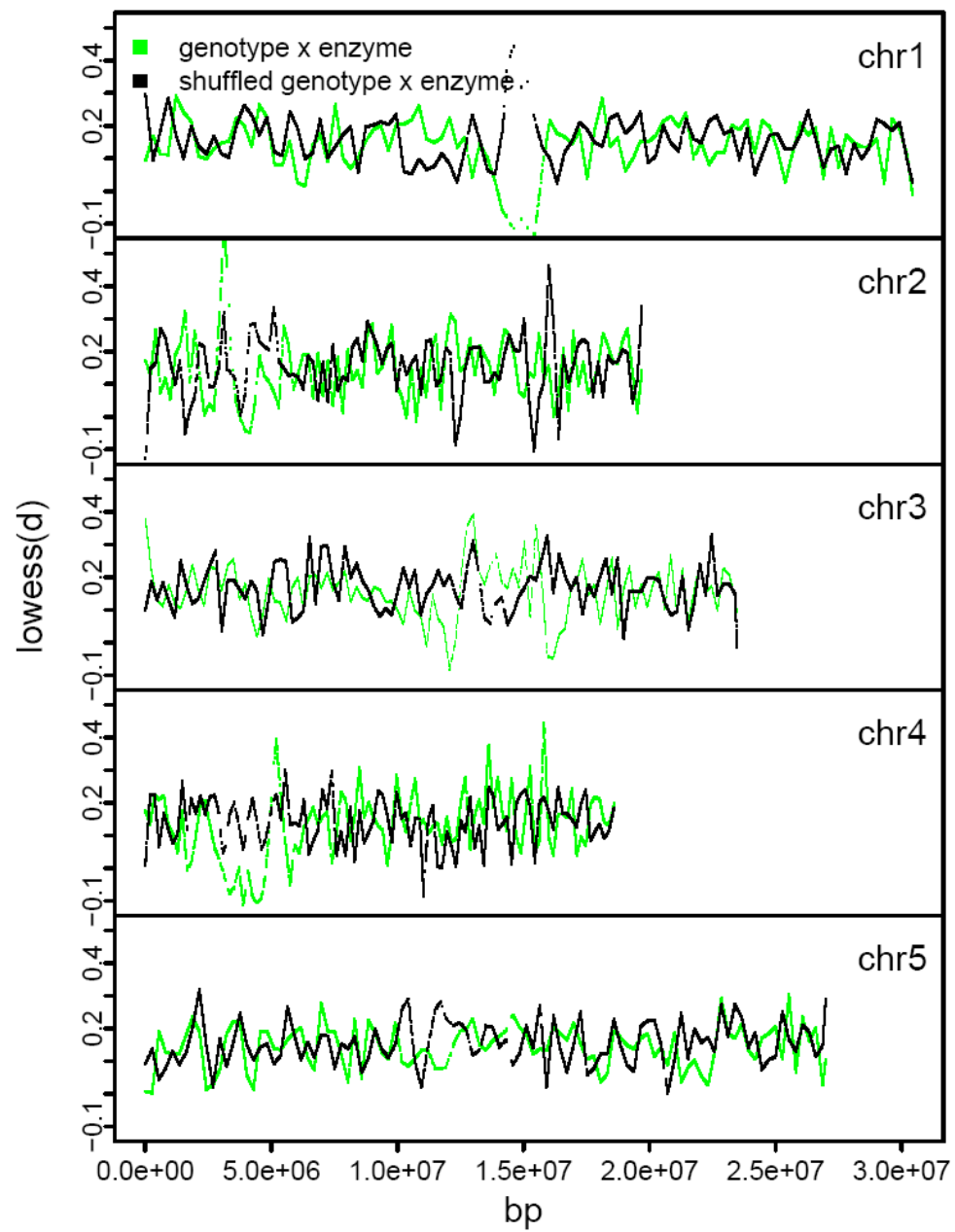

**B**

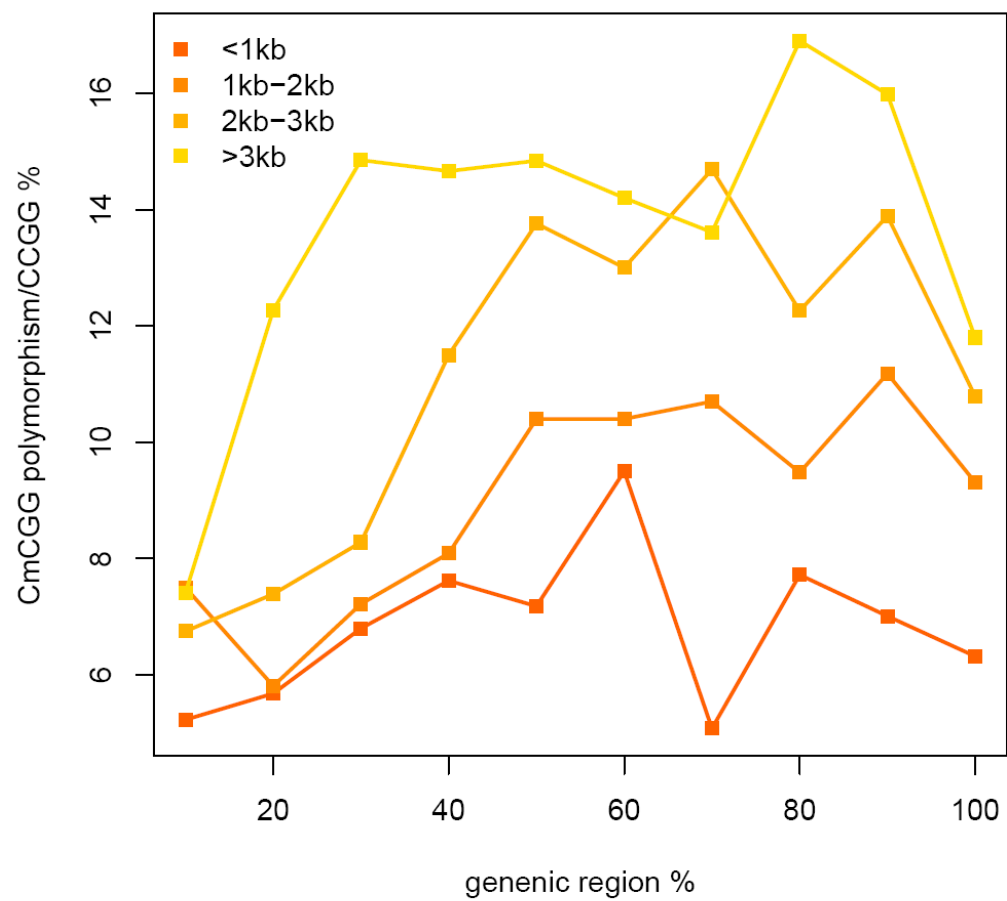

Supplement: Figure S5 — Distribution of Polymorphic CG Methylation Sites. (A) Co-regulation of methylation polymorphisms along chromosomes. The d scores of genotype × enzyme effect for 54,519 CCGG-containing features were Lowess-smoothed with a window size of 200 kb (green), or were shuffled by 1 kb block and Lowess-smoothed as a null distribution (black). The smoothed d scores (y-axis) were plotted along chromosome positions (x-axis). (B) Correlation between the size of gene (sequence flanked by UTRs) and the level of polymorphic CG methylation. Genes possessing CCGG-containing feature(s) were separated to 4 groups based on their size. Within each group, gene regions were divided to 10 percentiles based on position, and the percent polymorphic CG methylation (y-axis) was calculated for each percentile (x-axis). (0.08 MB PDF) [file pgen.1000032.s005.pdf]

A

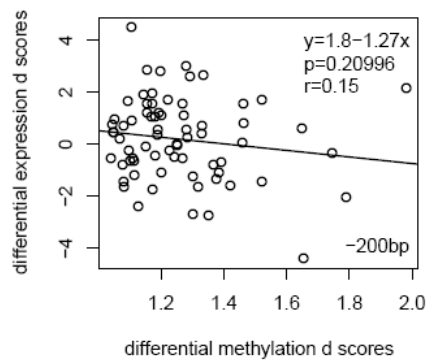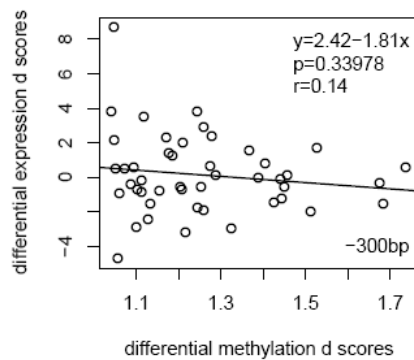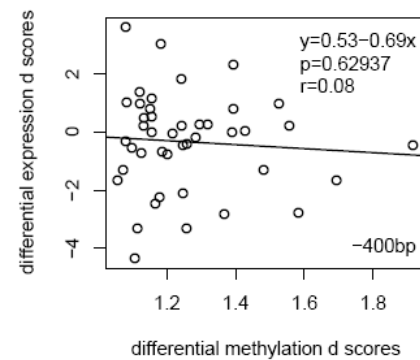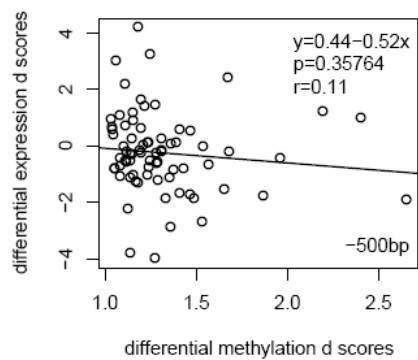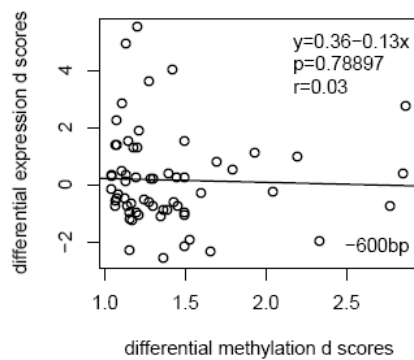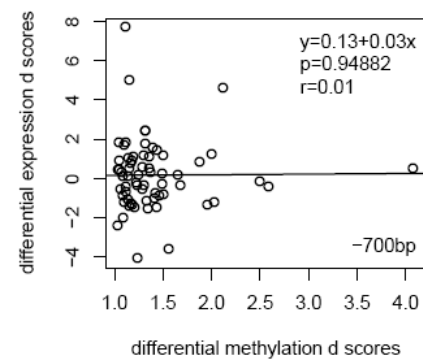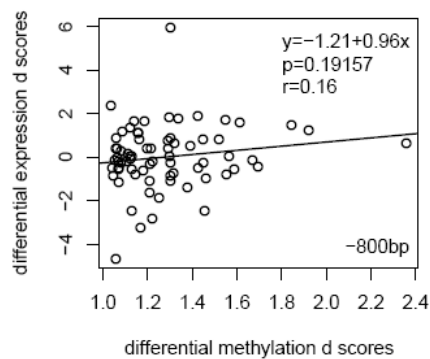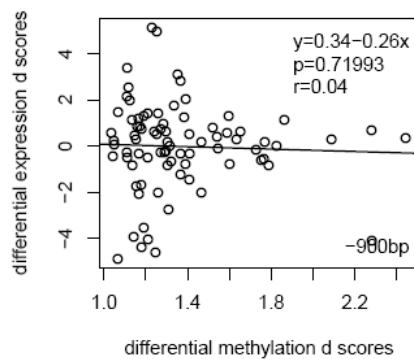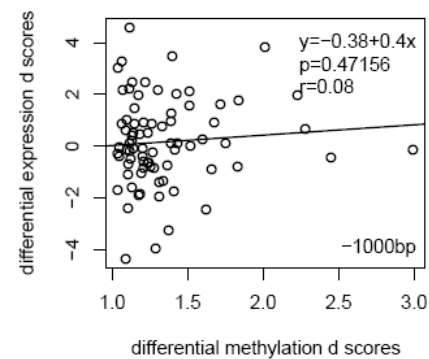

**B**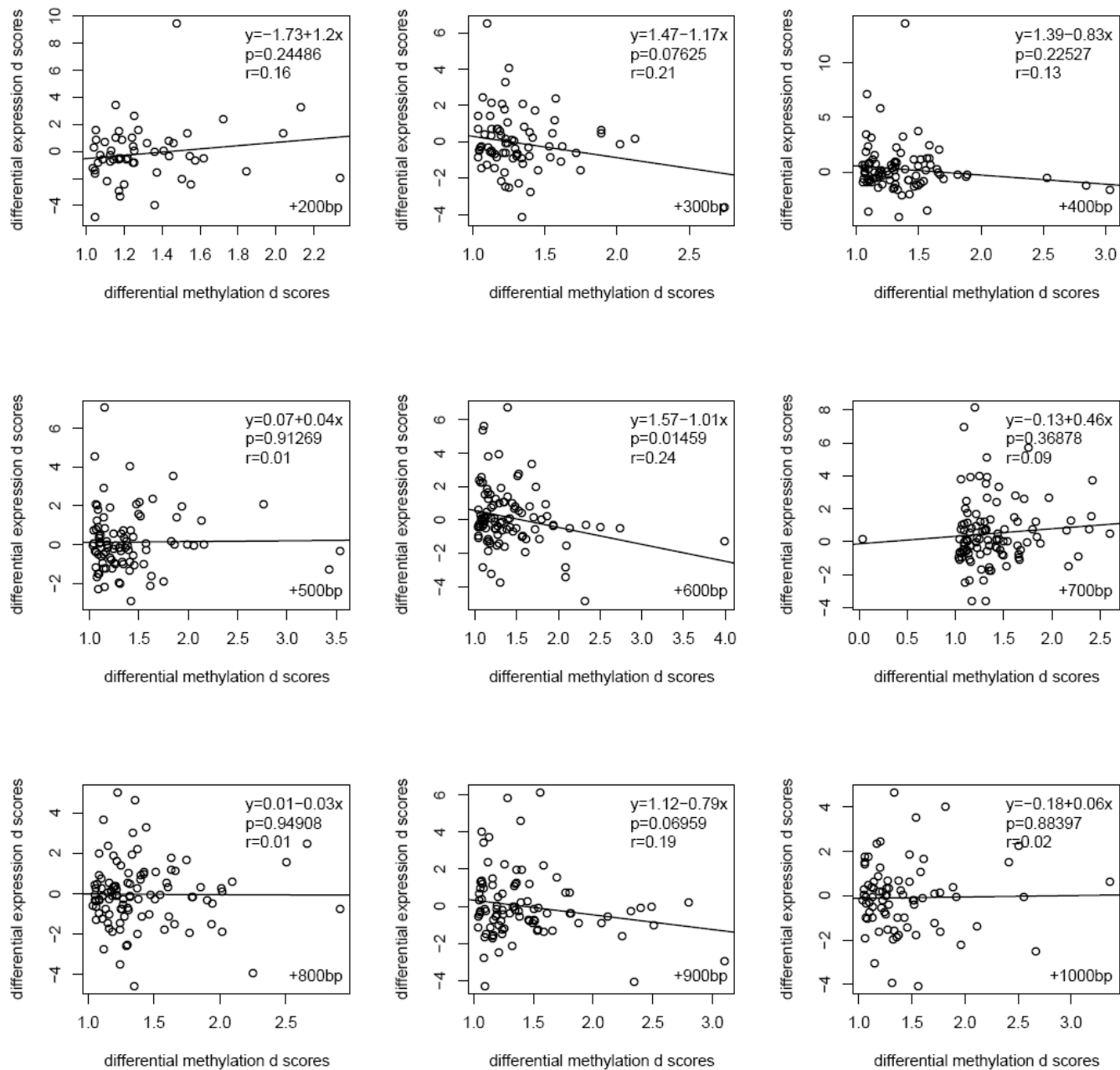

Supplement: Figure S8 — Correlation of Methylation Variation and Expression Variation. Differential gene expression d scores (y-axis) were linear regressed against polymorphic CG methylation d scores (x-axis) for features within (A) 100 bp intervals starting from upstream 100 bp to upstream 1000 bp; (B) 100 bp intervals starting from downstream 100 bp to downstream 1000 bp. (0.14 MB PDF) [file pgen.1000032.s008.pdf]
