# Supplementary material for: Global Analysis of Genetic, Epigenetic and Transcriptional Polymorphisms in Arabidopsis thaliana Using Whole Genome Tiling Arrays
Source: PLoS Genet. 2008 Mar 21;4(3):e1000032. doi: 10.1371/journal.pgen.1000032 (PMC2265482; doi:10.1371/journal.pgen.1000032)
Supplement: Figure S3 — Verification of Methylation or Methylation Polymorphisms by Quantitative Measurement. (A-C) Verification of methylation or methylation polymorphisms by epityper. The quantification scale of cytosine methylation was illustrated in the top panel. The CCGG site detected by microarray experiment was boxed. The corresponding gene and the relative position of the detected CCGG site within gene were illustrated in the bottom panel. For reciprocal hybrid lines, mother strain was listed first. Plant samples grown in two independent growth experiments were used in the epityper analysis. (A) polymorphic locus chr1/ 9491334; (B) polymorphic locus chr4/10420079; (C) constitutive locus chr1/22476369. (D-F) Verification of methylation polymorphisms by bisulfite sequencing. The percent methylation for all cytosine residues (y-axis) was plotted on their relative positions within the segment (x-axis). The CCGG site tested was pointed by the black arrow. (D) locus chr1/18407942; (E) locus chr1/27257856; (F) locus chr/30092640. (0.34 MB PDF) [file pgen.1000032.s003.pdf]

A

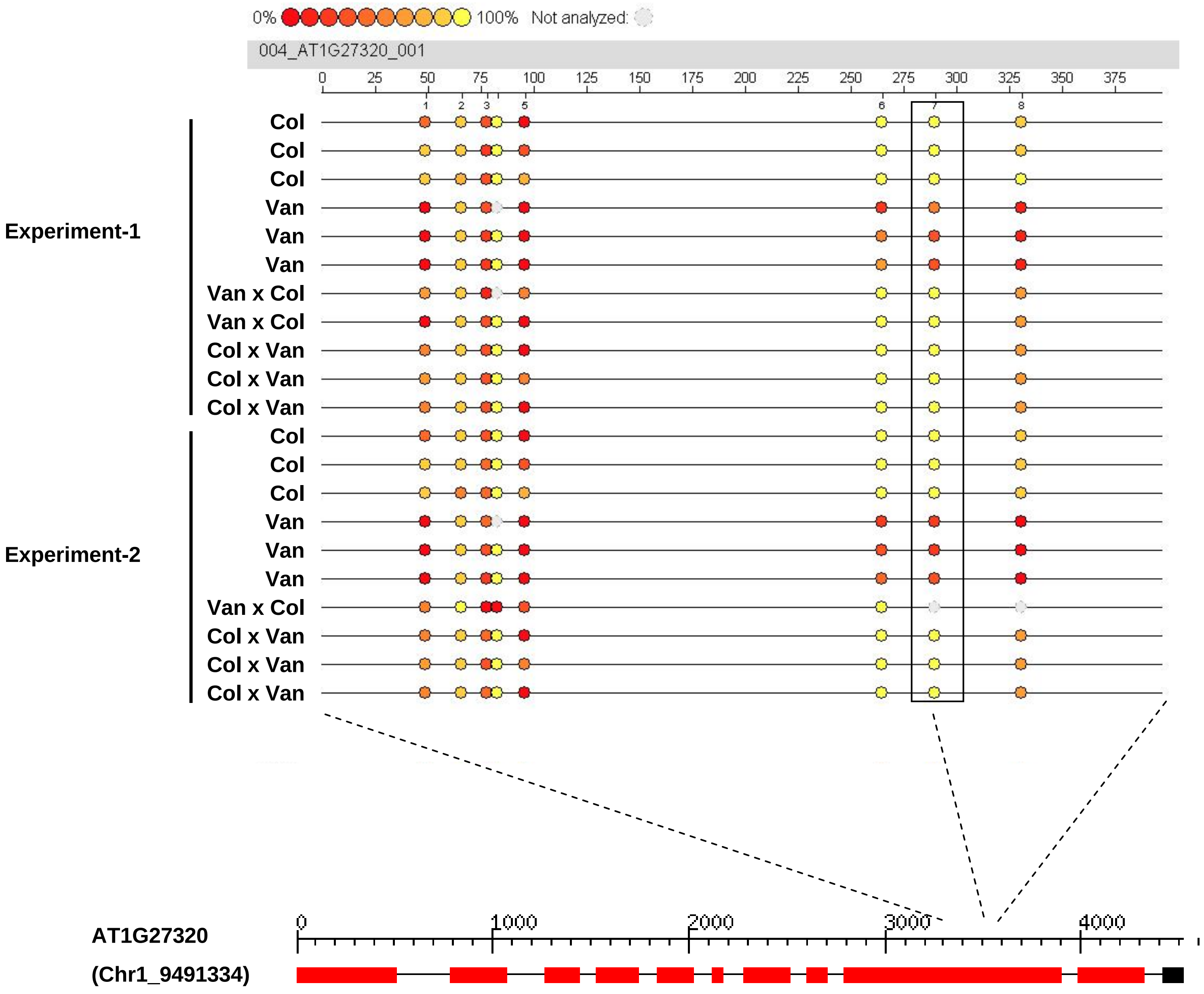
0%
100%
Not analyzed:
004_AT1G27320_001
0 25 50 75 100 125 150 175 200 225 250 275 300 325 350 375
1 2 3 5 6 7 8
Col
Col
Col
Van
Van
Van
Van x Col
Van x Col
Col x Van
Col x Van
Col x Van
Col
Col
Col
Van
Van
Van
Van x Col
Col x Van
Col x Van
Col x Van
Experiment-1
Experiment-2
AT1G27320
(Chr1_9491334)
0 1000 2000 3000 4000

B

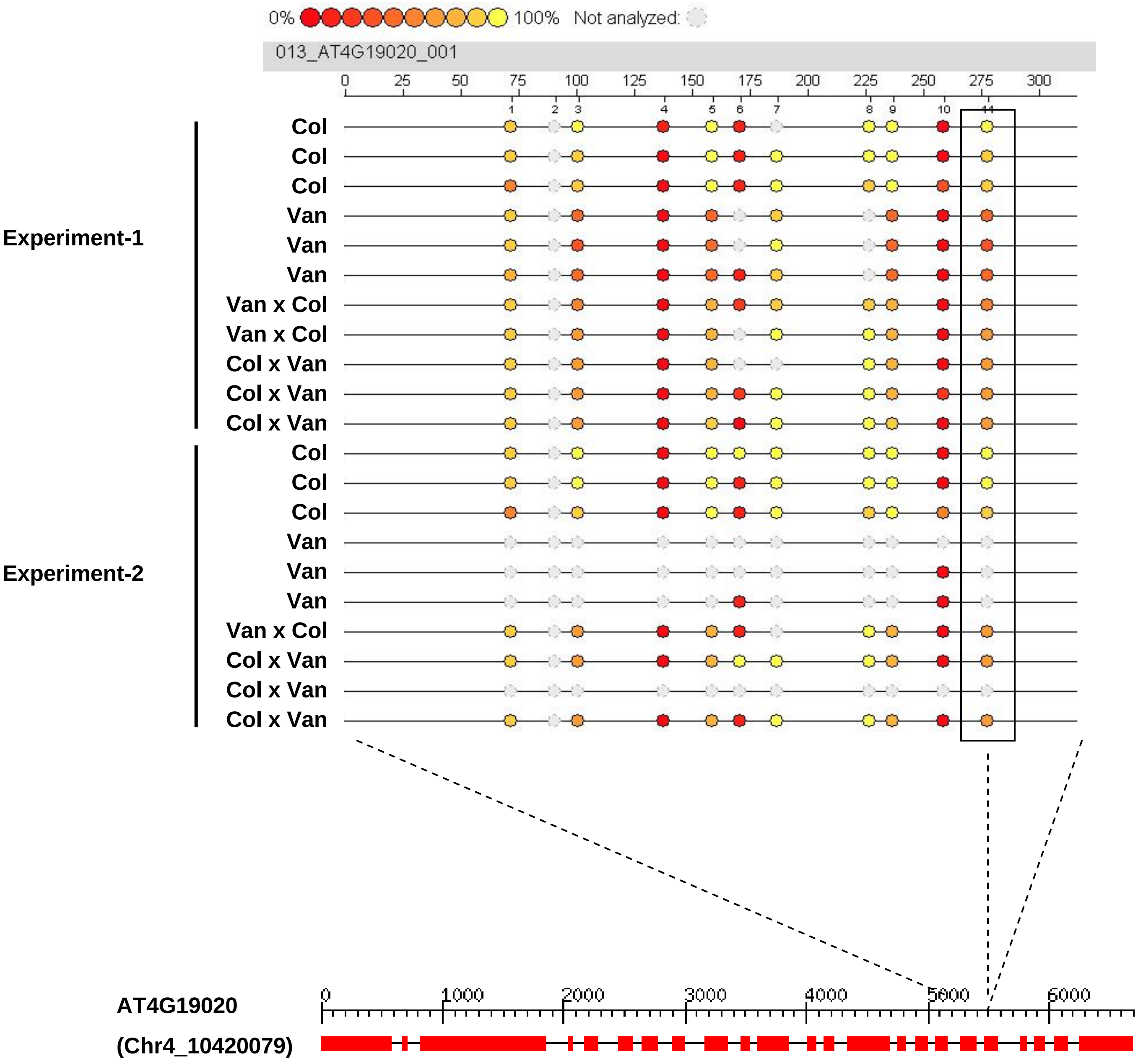

0%
100%
Not analyzed:
013_AT4G19020_001
0 25 50 75 100 125 150 175 200 225 250 275 300
1 2 3 4 5 6 7 8 9 10 11
Experiment-1
Col
Col
Col
Van
Van
Van
Van x Col
Van x Col
Col x Van
Col x Van
Col x Van
Experiment-2
Col
Col
Col
Van
Van
Van
Van x Col
Col x Van
Col x Van
Col x Van
AT4G19020
(Chr4_10420079)
0 1000 2000 3000 4000 5000 6000

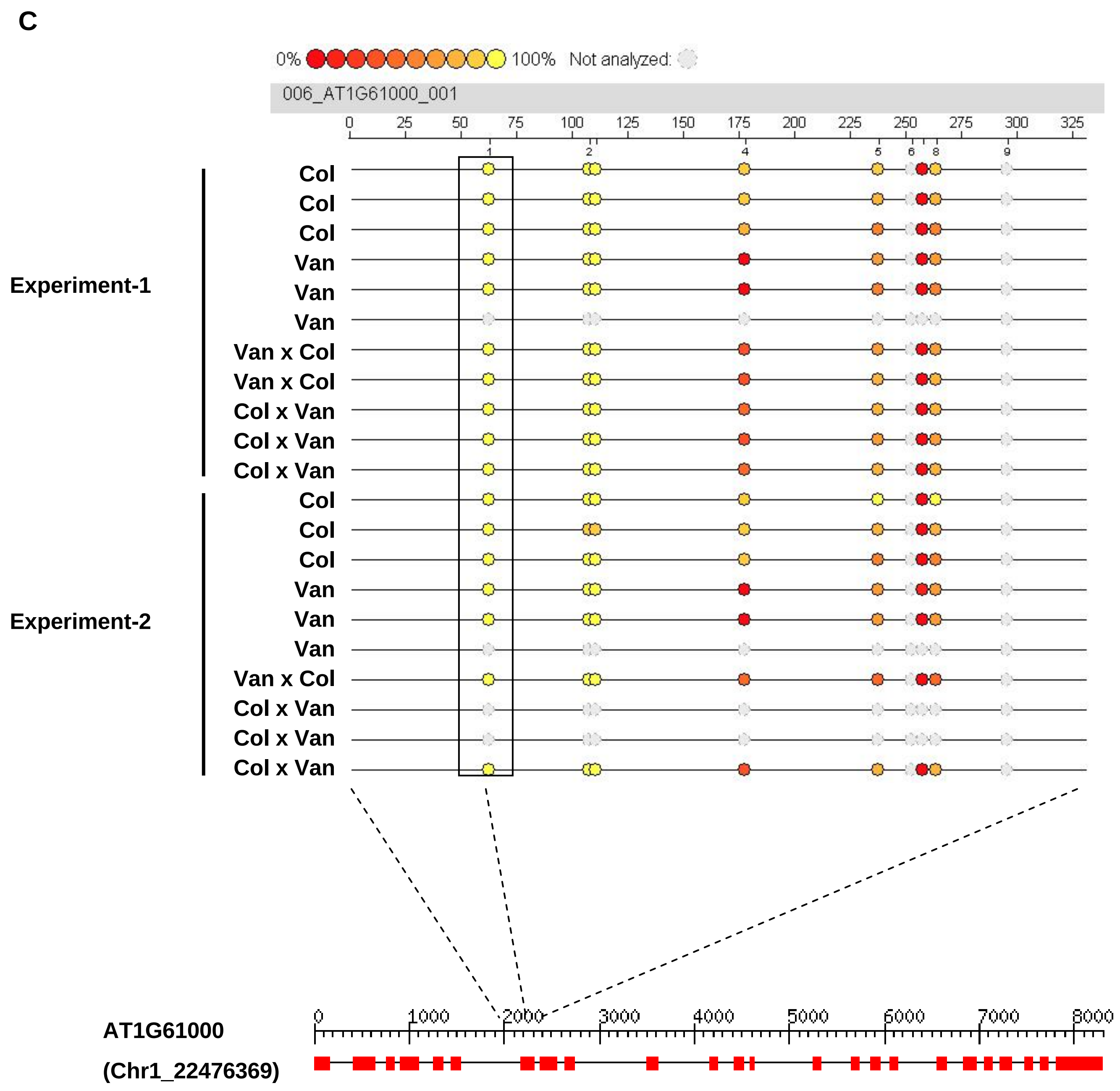
C
0%
100%
Not analyzed:
006_AT1G61000_001
0 25 50 75 100 125 150 175 200 225 250 275 300 325
1 2 4 5 6 8 9
Experiment-1
Col
Col
Col
Van
Van
Van
Van x Col
Van x Col
Col x Van
Col x Van
Col x Van
Experiment-2
Col
Col
Col
Van
Van
Van
Van x Col
Col x Van
Col x Van
Col x Van
AT1G61000
(Chr1_22476369)
0 1000 2000 3000 4000 5000 6000 7000 8000

D

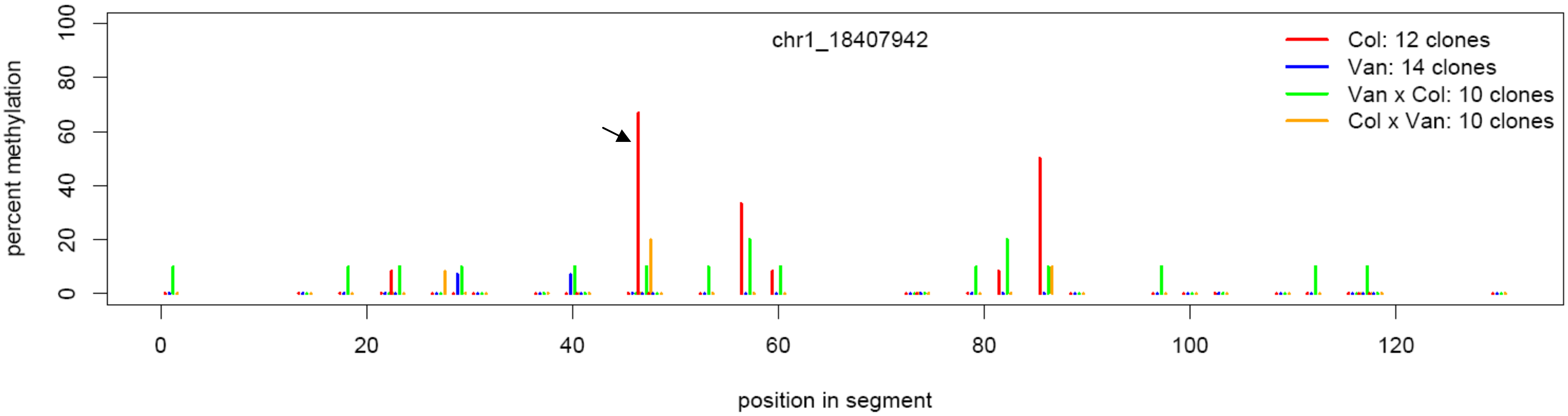
chr1_18407942
Col: 12 clones
Van: 14 clones
Van x Col: 10 clones
Col x Van: 10 clones
percent methylation
0
20
40
60
80
100
0
20
40
60
80
100
120
position in segment

E

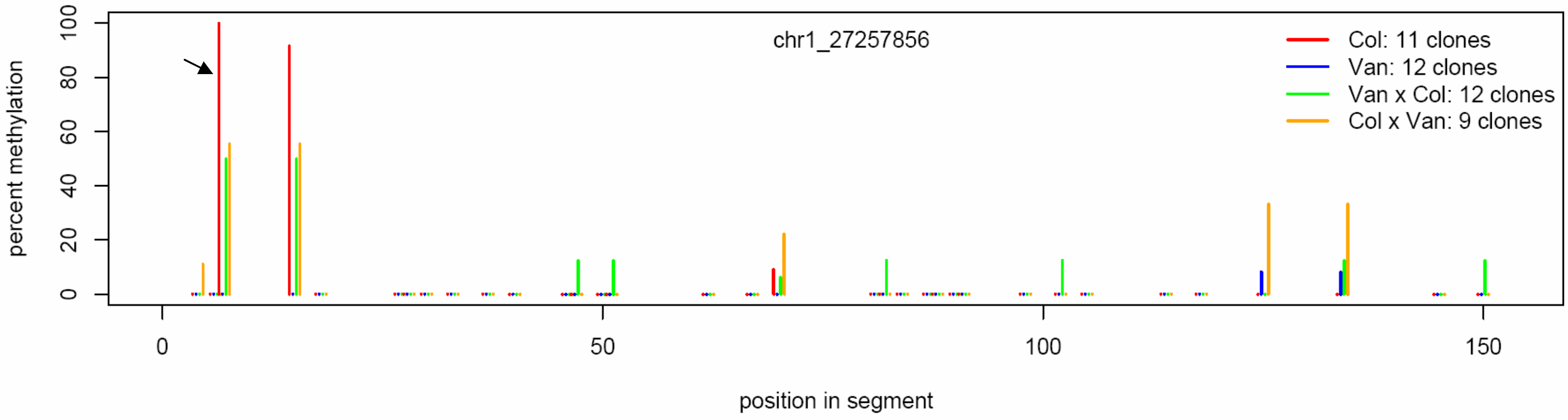

chr1_27257856
Col: 11 clones
Van: 12 clones
Van x Col: 12 clones
Col x Van: 9 clones
percent methylation
0
20
40
60
80
100
0
50
100
150
position in segment

F

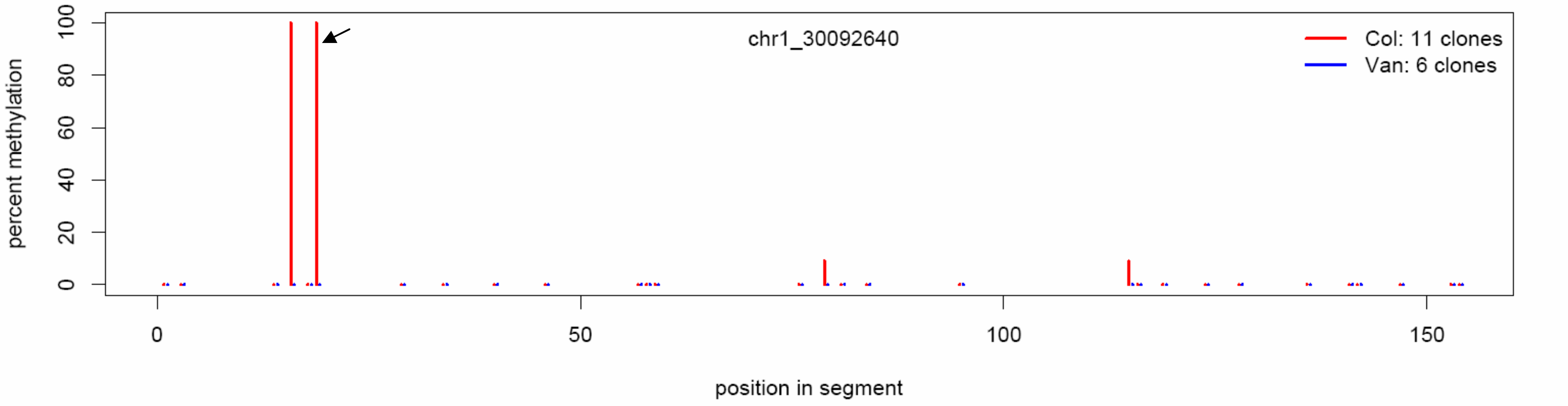
chr1_30092640
Col: 11 clones
Van: 6 clones
percent methylation
0
20
40
60
80
100
0
50
100
150
position in segment
