# Supplementary material for: Global Analysis of Genetic, Epigenetic and Transcriptional Polymorphisms in Arabidopsis thaliana Using Whole Genome Tiling Arrays
Source: PLoS Genet. 2008 Mar 21;4(3):e1000032. doi: 10.1371/journal.pgen.1000032 (PMC2265482; doi:10.1371/journal.pgen.1000032)
Supplement: Figure S6 — Diagram of each Effect in the (Additive+Dominant+Maternal) × Enzyme Full Model. F1c: F1 hybrids with Col-mother; F1v: F1 hybrids with Van-mother. Additive effect indicates hybridization intensity difference between parents across enzyme treatments, suggesting SFP. Dominant effect indicates hybridization intensity difference between the mid-parent (dashed line) and the average of reciprocal F1 hybrids across enzyme treatments. Maternal effect indicates hybridization intensity difference between reciprocal F1 hybrids across enzyme treatments. Enzyme effect with greater HpaII signal indicates constitutive CG methylation; that with greater MspI signal was likely due to normalization. Additive × enzyme effect indicates differential CG methylation between parents; dominant × enzyme effect indicates differential CG methylation between mid-parents and average of F1 hybrids; maternal × enzyme effect indicates differential CG methylation between reciprocal F1 hybrids. (0.10 MB PDF) [file pgen.1000032.s006.pdf]

Full model:

Intensity ~ add + dom + mat + enz + add x enz + dom x enz + mat x enz + ε

Contrast setting in the full model:

	add	dom	mat	enz
Col *Hpa*II	1	0	0	1
Col *Msp*I	1	0	0	-1
Van *Hpa*II	-1	0	0	1
Van *Msp*I	-1	0	0	-1
F1c *Hpa*II	0	1	1	1
F1c *Msp*I	0	1	1	-1
F1v *Hpa*II	0	1	-1	1
F1v *Msp*I	0	1	-1	-1

Add: additive effect; dom: dominant effect; mat: maternal effect; enz: enzyme effect; ε: random error.

Additive effect +

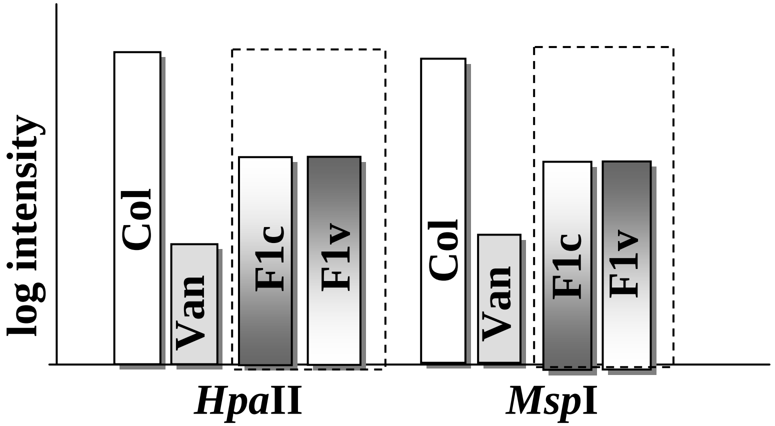

SFP; Col has greater signal than Van.

Additive effect -

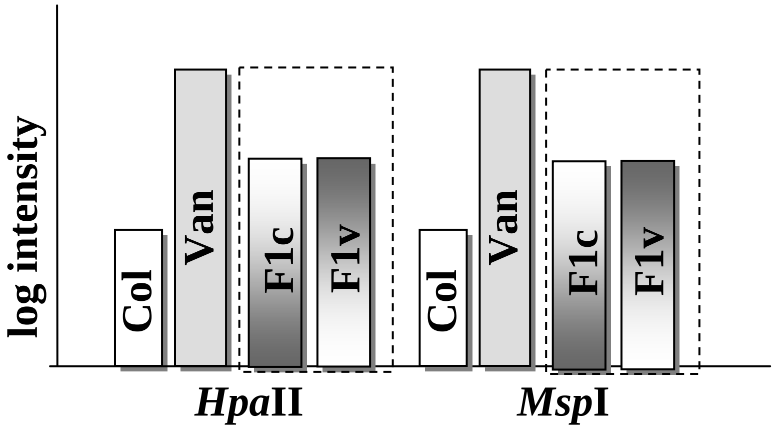

Van duplication or deletion in Col; Van has greater signal than Col

Additive effect describes intensity difference between parent strains across enzyme treatments.

Dominant effect +

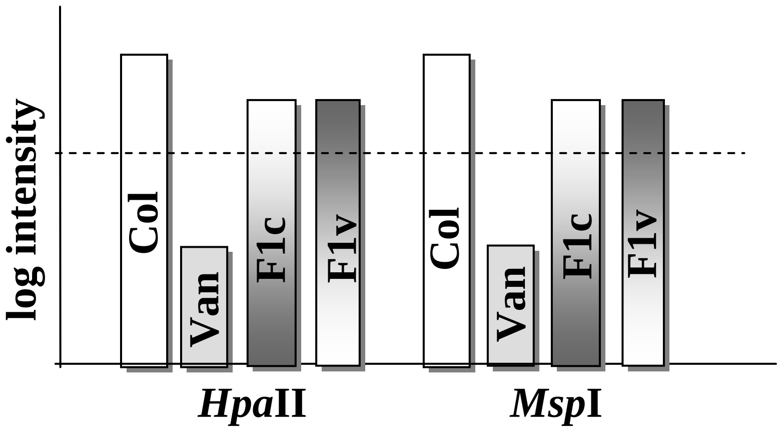

Increased F1 hybridization compared with expected from mid-parent

Dominant effect -

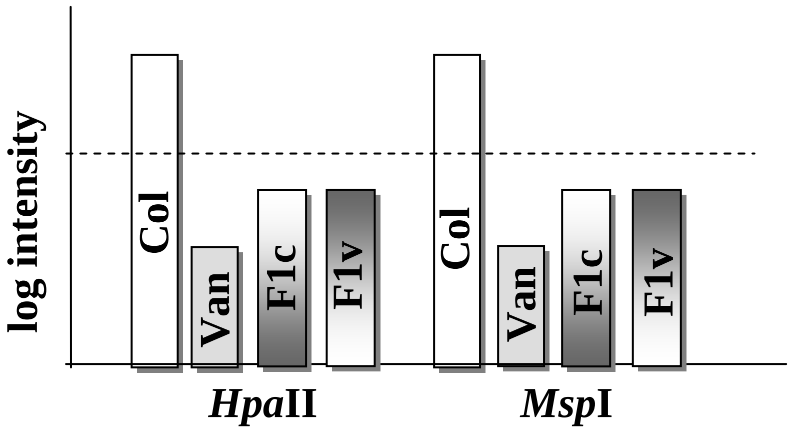

Reduced F1 hybridization compared with expected from mid-parent

Dominant effect describes intensity difference between mid-parent (average of parents; dashed line) and average of F1 hybrids across enzyme treatments.

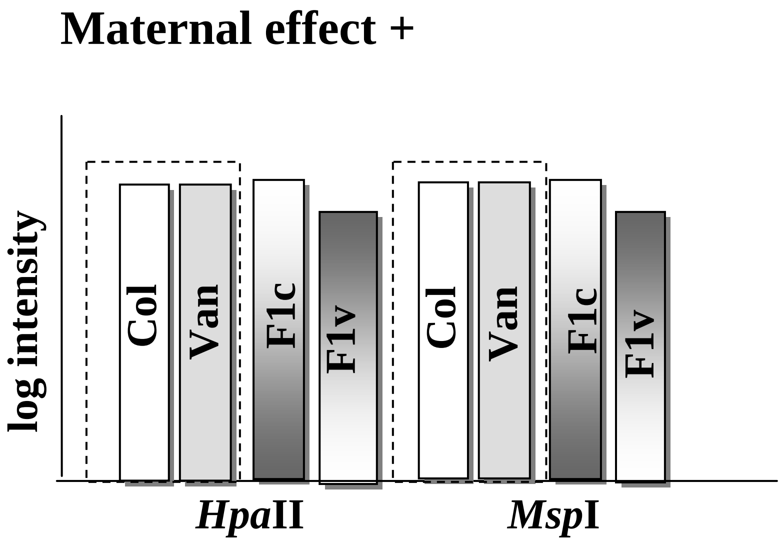

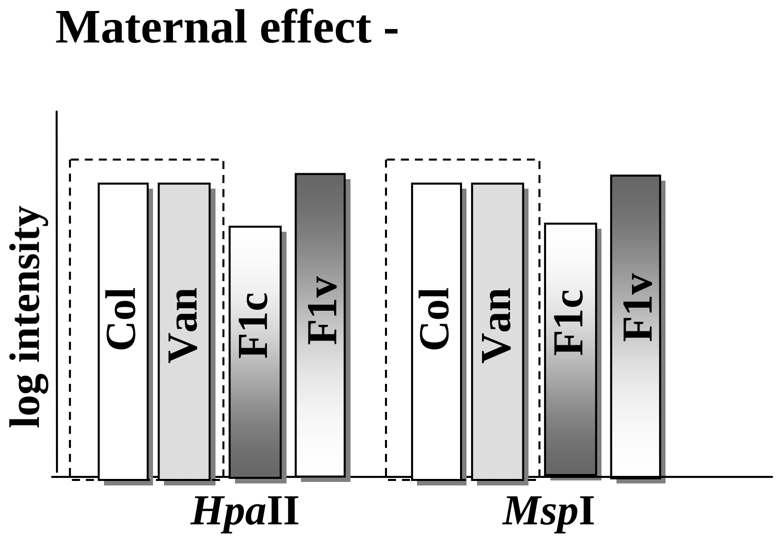

Random variation; Col-mother F1 with greater signal than Van-mother F1

Random variation; Van-mother F1 with greater signal than Col-mother F1

Maternal effect describes intensity difference between reciprocal F1 hybrids across enzyme treatments.

Enzyme effect +

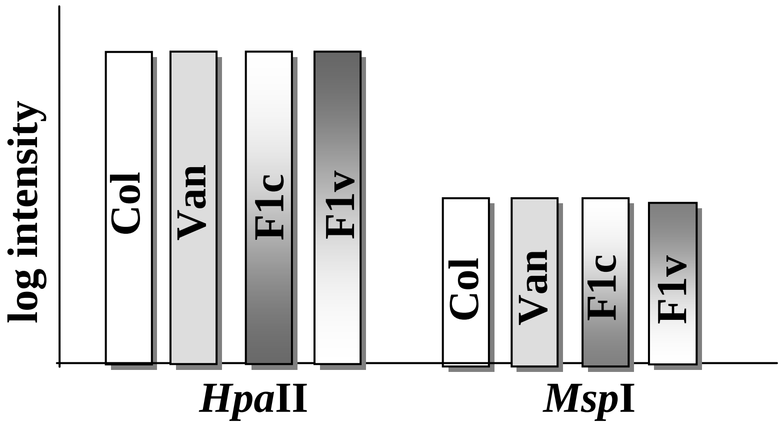

Constitutive CG methylation; *Hpa*II samples have greater signal

Enzyme effect -

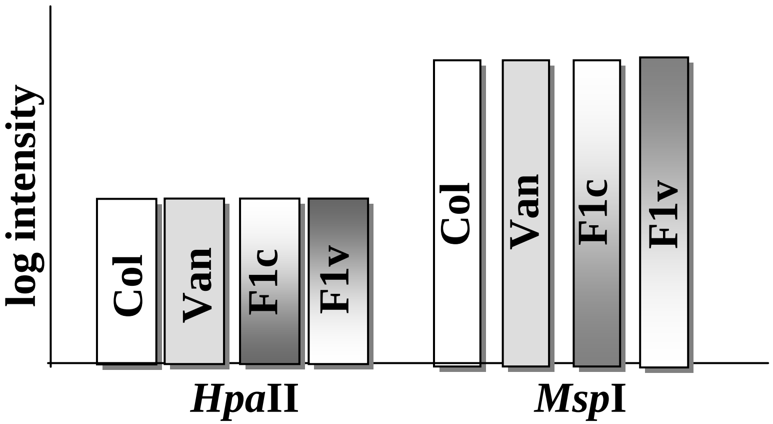

Normalization; *Msp*I samples have greater signal

Enzyme effect describes intensity difference between *Hpa*II and *Msp*I enzyme treatment across genotypes.

Additive x enzyme effect +

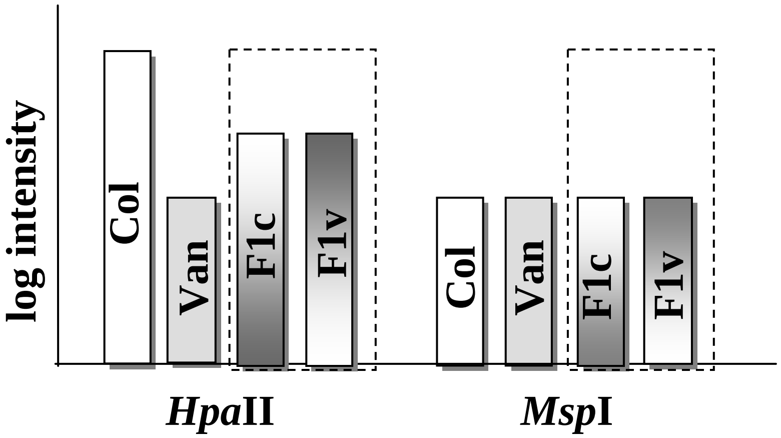

Col-specific methylation

Additive x enzyme effect -

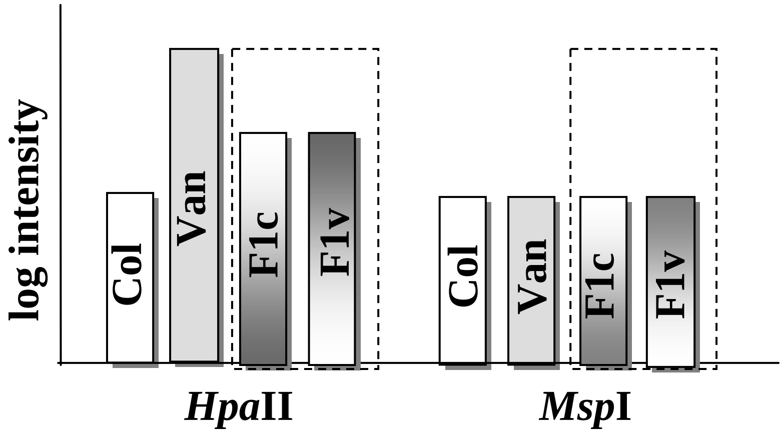

Van-specific methylation

Additive x enzyme effect describes differential enzyme sensitivity between parent strains.

Dominant x enzyme effect +

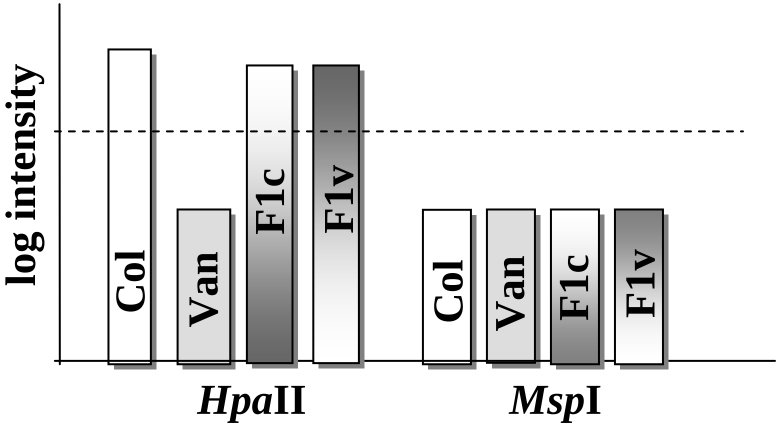

Col-dominant methylation

Dominant x enzyme effect -

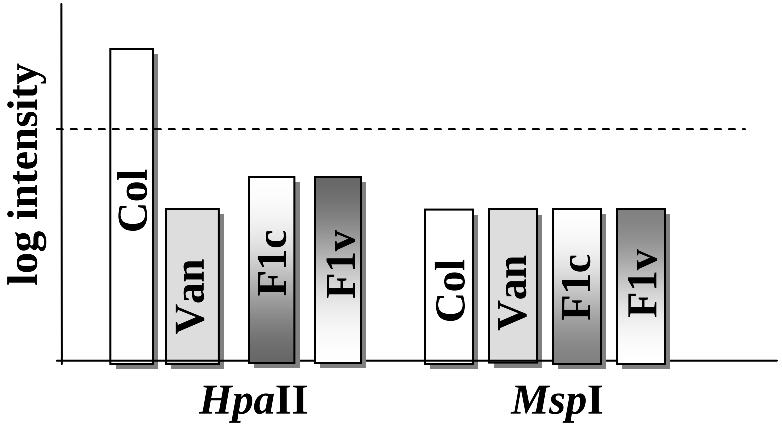

Van-dominant methylation

Dominant x enzyme effect describes differential enzyme sensitivity between mid-parent (average of parents; dashed line) and average of F1 hybrids.

Maternal x enzyme effect +

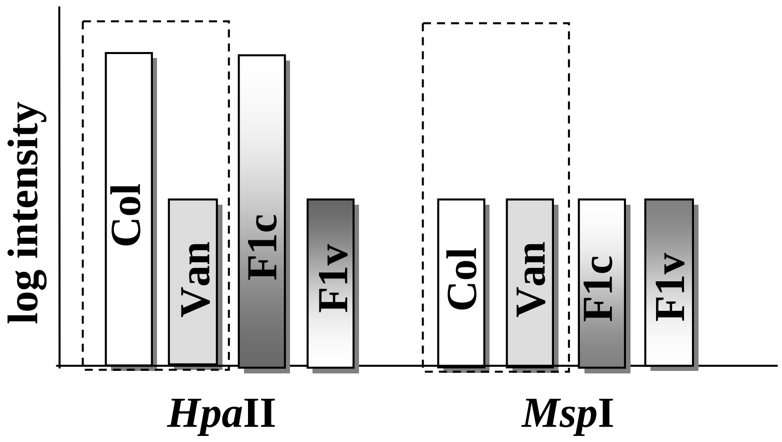

Col-mother hybrid specific methylation

Maternal x enzyme effect -

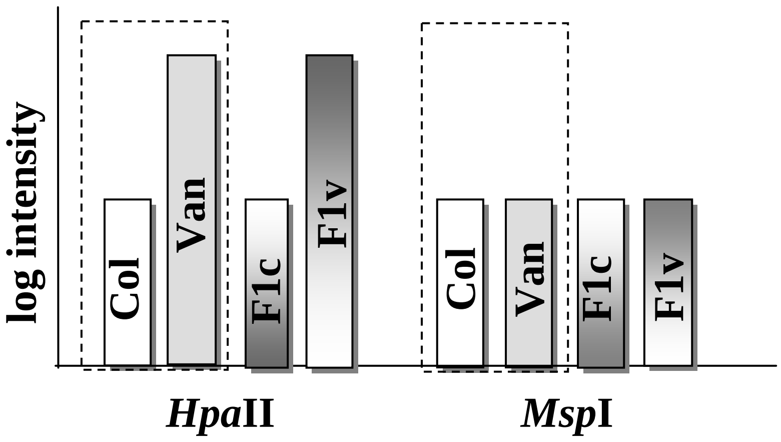

Van-mother hybrid specific methylation

Maternal x enzyme effect describes differential enzyme sensitivity between reciprocal F1 hybrids
